# Endocytosis of the Anthrax Toxin Is Mediated by Clathrin, Actin and Unconventional Adaptors

**DOI:** 10.1371/journal.ppat.1000792

**Published:** 2010-03-05

**Authors:** Laurence Abrami, Mirko Bischofberger, Béatrice Kunz, Romain Groux, F. Gisou van der Goot

**Affiliations:** Global Health Institute, Ecole Polytechnique Fédérale de Lausanne, Faculty of Life Sciences, Lausanne, Switzerland; The Salk Institute for Biological Studies, United States of America

## Abstract

The anthrax toxin is a tripartite toxin, where the two enzymatic subunits require the third subunit, the protective antigen (PA), to interact with cells and be escorted to their cytoplasmic targets. PA binds to cells via one of two receptors, TEM8 and CMG2. Interestingly, the toxin times and triggers its own endocytosis, in particular through the heptamerization of PA. Here we show that PA triggers the ubiquitination of its receptors in a β-arrestin-dependent manner and that this step is required for clathrin-mediated endocytosis. In addition, we find that endocytosis is dependent on the heterotetrameric adaptor AP-1 but not the more conventional AP-2. Finally, we show that endocytosis of PA is strongly dependent on actin. Unexpectedly, actin was also found to be essential for efficient heptamerization of PA, but only when bound to one of its 2 receptors, TEM8, due to the active organization of TEM8 into actin-dependent domains. Endocytic pathways are highly modular systems. Here we identify some of the key players that allow efficient heptamerization of PA and subsequent ubiquitin-dependent, clathrin-mediated endocytosis of the anthrax toxin.

## Author Summary

*Bacillus anthracis* is the bacterium responsible for the anthrax disease. Its virulence is mainly due to 2 factors, the anthrax toxin and the anti-phagocytic capsule. This toxin is composed of three independent polypeptide chains. Two of these have enzymatic activity and are responsible for the effects of the toxin. The third has no activity but is absolutely required to bring the 2 enzymatic subunits into the cell where they act. If one blocks entry into the cells, one blocks the effects of these toxins, which is why it is important to understand how the toxin enters into the cell at the molecular level. Here we identified various molecules that are involved in efficiently bringing the toxin into the cell. First, we found that the actin cytoskeleton plays an important role in organizing one of the two anthrax toxin receptors at the cell surface. Second, we found a cytosolic protein, β-arrestin, that is required to modify the intracellular part of the toxin receptor, to allow uptake. Finally, we directly show, for the first time, that anthrax toxin uptake is mediated by the so-called clathrin-dependent pathway, a very modular entry pathway, but that the toxin utilizes this pathway in an unconventional way.

## Introduction

Bacterial toxins endowed with enzymatic activity generally have targets, or require co-factors, that reside in the cytoplasm of the target cell. Such is the case for the anthrax toxin produced by *Bacillus anthracis*. It is composed of three independent polypeptide chains, 2 of which have an enzymatic activity–edema factor (EF) and lethal factor (LF)–and one, the protective antigen (PA), which has the ability to interact with the target cell. EF is a calmodulin dependent adenylate cyclase that must thus reach the cytoplasm to become active. LF is a metalloprotease that cleaves MAP kinase kinases, all of which are cytoplasmic [1],[2].

EF and LF reach their final destination through the help of PA. PA binds specifically to two identified anthrax toxin receptors, tumor endothelial marker 8 (TEM8, also called ANTXR1) and capillary morphogenesis 2 (CMG2, or ANTXR2), two type I membrane proteins that share extensive sequence similarity both in their extracellular and intracellular domains [3],[4]. PA, which is produced by the bacterium as an 83 kDa form (PA83), is processed into an 63 kDa form, by host enzymes such as the endoprotease furin [5]. The thus generated PA63 has the capacity to heptamerize (PA^7mer^) into a ring-like structure, which serves as the receptor of EF and LF [6]. The hetero oligomeric complex–i.e. PA^7mer^-EF/LF and receptors–is then internalized by the cell and delivered to early endosomes, where PA^7mer^ undergoes a conformational change that leads to its membrane insertion and pore-formation (pPA^7mer^) [2]. EF and LF are also sensitive to the acidic pH of endosomes, which leads to their partial unfolding, allowing them to be translocated through the lumen of the PA channel to the other side of the membrane [7].

Considering that PA^7mer^ is the receptor for EF and LF, one crucial point is that PA should not undergo endocytosis in its monomeric form, i.e. the enzymatic subunits would fail to be delivered to the cytoplasm. This is indeed what has been observed by us and others [8],[9] leading to the notion that the anthrax toxin times its entry into the cells, in particular by triggering the activation of *src*-like kinases [10].

We have previously shown that processing of PA leads to a relocalization of the toxin from the glycerolipid region of the plasma membrane to lipid rafts [8], where the receptors encounter the E3 ubiquitin ligase Cbl that modifies a juxtamembranous lysine of the cytoplasmic tail of the receptors [11]. Ubiquitination in turn promotes endocytosis of the toxin through a mechanism that requires the large GTPase dynamin [8],[12]–involved in vesicles fission, but not the caveolar proteins caveolin-1, pointing towards a role for clathrin in anthrax toxin endocytosis [8].

We here sought to identify other molecular players involved in anthrax toxin uptake. We show that clathrin is, as predicted, involved in endocytosis but that the anthrax toxin follows a non-canonical clathrin-dependent route that depends of β-arrestins and the heterotetrameric adaptor complex AP-1. Moreover we show that endocytosis is strongly actin dependent, in contrast to endocytosis of another bacterial toxin, diphtheria toxin. Interestingly we found that actin also promotes the heptamerization of PA63, but only when it is bound to TEM8, not when bound to CMG2.

## Results

### Endocytosis of the anthrax toxin is clathrin mediated

Previous studies of our laboratory indicated that anthrax toxin endocytosis in HeLa cells is independent of caveolin-1 but was affected by the overexpression of dominant negative mutants of dynamin 2 or of the accessory protein Eps15 [8]. Here we sought to confirm these findings using an independent method, namely gene silencing by RNAi, to more precisely define the molecular players involved in anthrax toxin endocytosis.

**Figure 1:**
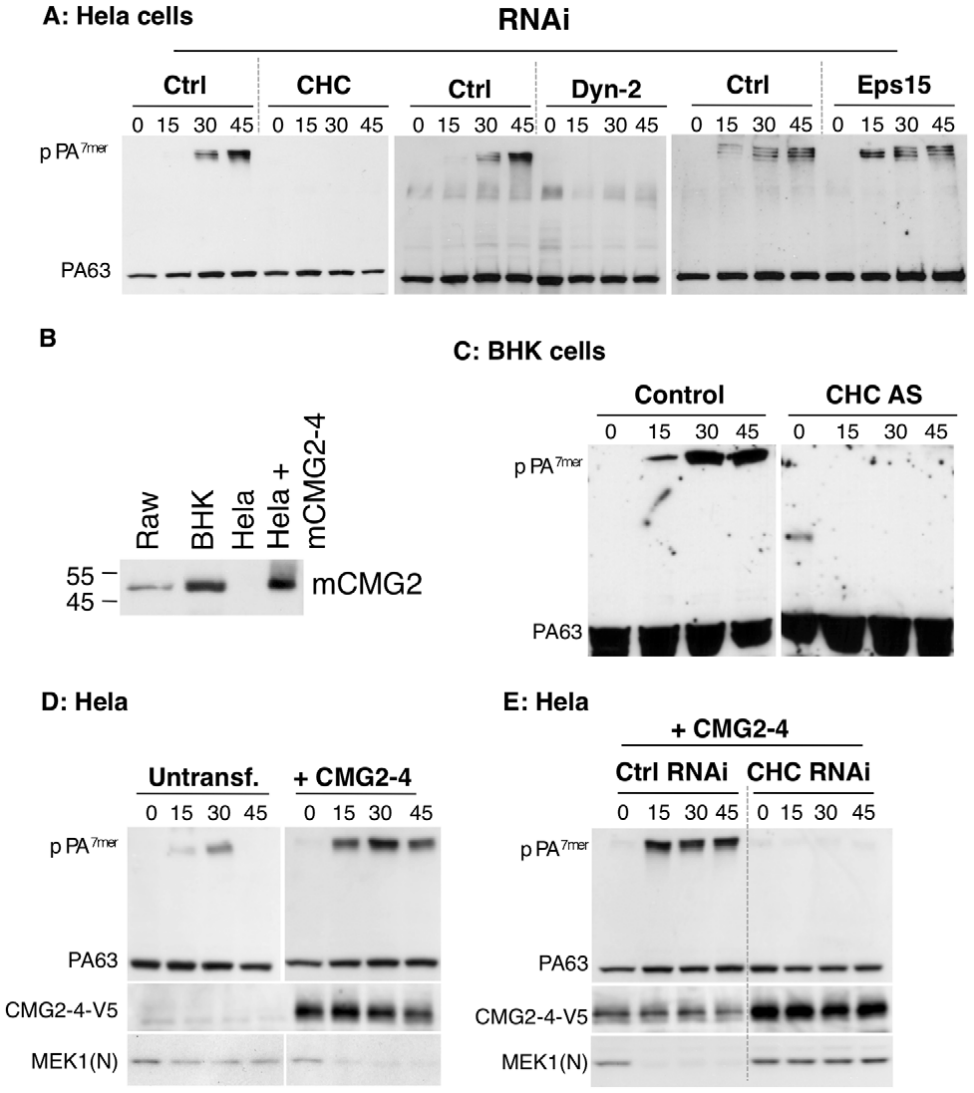
Anthrax toxin enter cells by clathrin mediated endocytosis. A: Hela cells were transfected 72 hrs with control siRNAs or siRNAs against Clathrin Heavy Chain (CHC), the dynamin 2 (Dyn-2) or Epidermal growth factor receptor pathway substrate 15 (Eps 15). Cells were incubated with 500 ng/ml PA63 for 1 hr at 4°C and different times at 37°C and cell extracts (40 µg of proteins) were analyzed by SDS-PAGE and western blotting to reveal PA monomer and SDS-resistant heptamer (pPA^7mer^). B: Cell extracts (40 µg of protein) from Raw, BHK and Hela–transfected or not with mouse CMG2-4–cells were analyzed by SDS-PAGE and Western blotting for the expression of CMG2. C: Stable BHK21-tTA/anti-CHC cells maintained in tetracycline (control cells) or not (CHC AS) were incubated with 500 ng/ml PA63 for 1 hr at 4°C and different times at 37°C and extracts (40 µg of proteins) were analyzed by SDS-PAGE and western blotting to reveal PA63 and SDS-resistant heptamer (pPA^7mer^). DE: Hela cells were transfected 72 hrs with human CMG2-V5 and with control siRNAs or siRNAs against CHC. Cells were then treated as in A. Cells extracts (40 µg of proteins) were analyzed by SDS-PAGE and western blotting to reveal the different forms of PA, CMG2-V5 and the N-terminus of the LF target MEK1 (MEK1 (N)).

As mentioned, PA63 heptamerizes at the cell surface in to PA^7mer^, which is an SDS-sensitive complex. Upon arrival in endosomes, PA^7mer^ converts to an SDS-resistant form (pPA^7mer^), which is transmembrane. Although formation of pPA^7mer^ is a late event, which occurs only after sorting of the toxin-receptor complex into the intraluminal vesicles of multivesicular endosomes [13], monitoring formation of pPA^7mer^ as a function of time is a convenient read-out to identify factors that affect toxin endocytosis. As shown in Fig. 1A, RNAi against either CHC or dynamin 2 strongly delayed the formation of the pPA^7mer^ in HeLa cells, whereas RNAi against Eps15 had no effect, despite efficient silencing of protein expression (Fig. S1A). We believe that the discrepancy between our present findings based on RNAi and our previous conclusions based on over-expression of dominant negative Eps15 is due to the fact that inhibition of either transferrin or PA endocytosis required strong over-expression, which might have been somewhat toxic [8]. Moreover, the effect of Eps15 over-expression was by no means as strong as the one observed for dynamin, in parallel experiments [8]. Hela cells express mainly TEM8. The above experiments therefore indicate that PA is internalized in a clathrin dependent manner when bound to TEM8. To investigate whether CMG2-mediated PA uptake is also clathrin dependent, we first made use of Baby Hamster Kidney (BHK) cells–which strongly express CMG2 (Fig. 1B) but have undetectable levels of TEM8 messenger (not shown)–and for which a cell line is available that allows the inducible expression of clathrin heavy chain (CHC) antisense RNA [14]. Formation of pPA^7mer^ was strongly delayed in CHC antisense RNA-expressing BHK cells (Fig. 1C). Since formation of pPA^7mer^ is a rather late event, we used a recently established FACS-based assay to monitor the initial step of endocytosis of the toxin [10]. PA was pre-bound to BHK cells at 4°C. Cells were then incubated at 37°C for different times prior to PA labeling at 4°C and FACS analysis. Incubation at 37°C led to a decrease of surface fluorescence for control cells, indicating that PA had been internalized but not for the CHC antisense RNA-expressing BHK cells (Fig. S1B).

To independently confirm the involvement of clathrin in CMG2-mediated PA endocytosis, we ectopically expressed CMG2 in Hela cells and performed RNAi against CHC. As shown in Fig. 1D, ectopic expression of CMG2 markedly accelerated and increased the formation of pPA^7mer^, indicating that the transfected receptor was mediating PA uptake. This was confirmed by the fact that immunoprecipitation of V5-tagged CMG2 led to the co-immunoprecipitation of PA63 and pPA^7mer^ (Fig. S1C, left panel). We next performed RNAi against CHC in these cells and found that formation of pPA7mer was abolished during the first 45 min (Fig. 1E), indicating that CHC was implicated not only in TEM8 mediated PA uptake but also CMG2-mediated uptake.

### Clathrin mediated toxin uptake is AP1 and β-arrestin dependent

Clathrin coated vesicles are composed of three layers: the inner layer consists of the cargo, the outer layer is composed by clathrin and the middle layer is composed of adaptor and accessory/regulatory molecules that link the cargo to the clathrin coat [15]. We here investigated the possible involvement of the heterotetrameric adaptor complex AP-2–arguably the most widely used adaptor in the formation of clathrin-coated vesicles at the plasma membrane [16]–, of AP-1–which mostly operates on the Trans-Golgi Network and endosomes–, and the adaptor proteins Grb2, Dab2 and β-arrestins. β-arrestins have been shown to act as adaptors, linking cargo receptors to clathrin, either directly, or via AP2 [17]. Their involvement has been shown in particular during the endocytosis of G-coupled receptors but also single-transmembrane receptors, such as the tyrosine kinase IGF-1R [18] or the Drosophila Notch receptor [19].

Expression of these different adaptors/accessory proteins was silenced by RNAi, and formation of pPA^7mer^, the SDS-resistant heptameric PA pore, was monitored as a function of time. As a second read-out, we also monitored the cleavage of the LF target MEK1, which reveals the delivery of LF to the cytoplasm. RNAi against CHC and the E3 ligase Cbl were used as positive controls. All RNAi duplexes were efficient at lowering the expression levels of the corresponding proteins (Fig. S1A). Due to the high degree of similarity between β-arrestin-1 and 2, silencing one gene led to a decrease in the levels of the other. Our studies can therefore not discriminate between the two forms.

**Figure 2:**
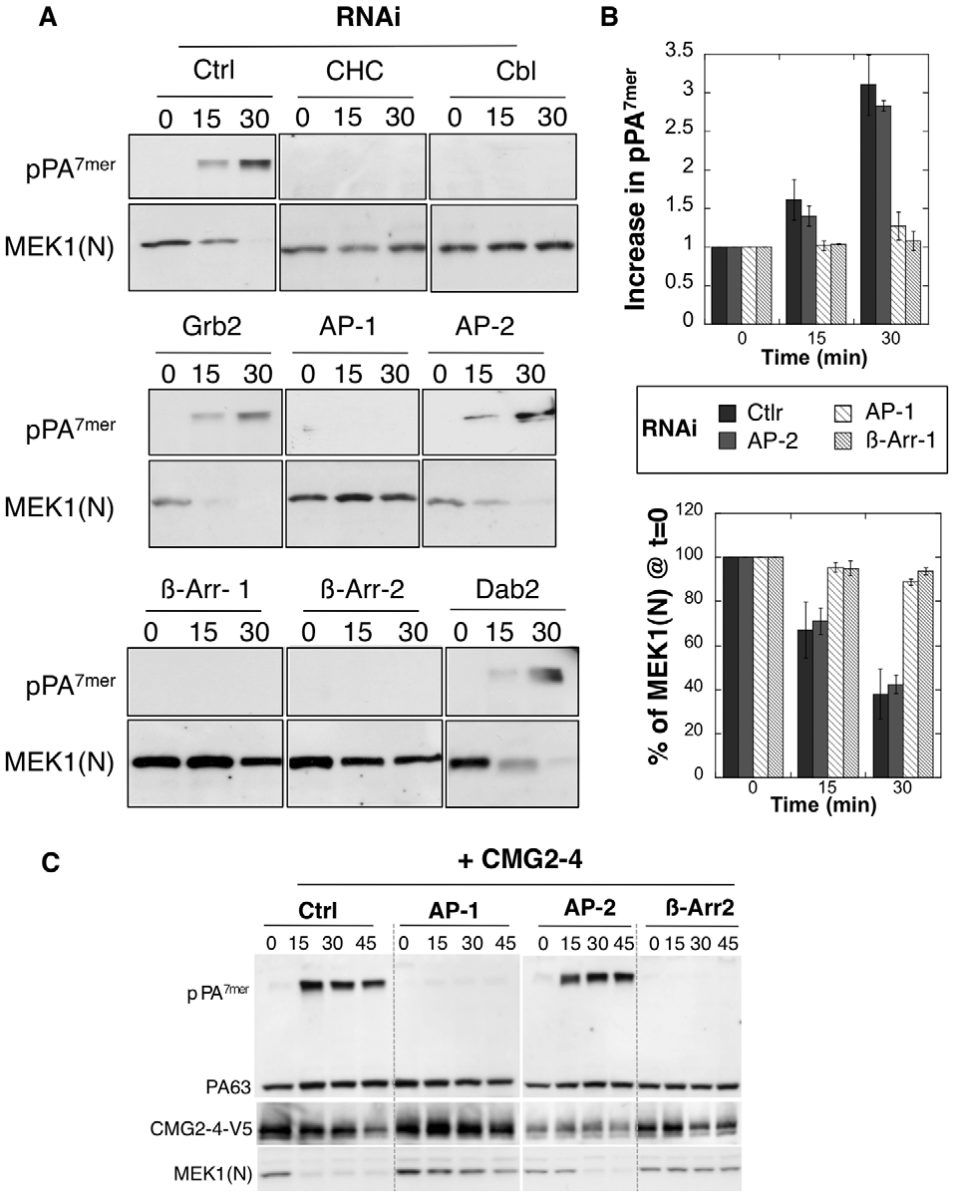
Endocytosis of PA is β-arrestin and AP-1 dependent. A-B: Hela cells were transfected 72 hrs with control siRNAs or siRNAs against Clathrin Heavy Chain (CHC), the ubiquitin ligase E3 Cbl (Cbl), Grb2, AP-1, AP-2, β-arrestin-1 (β-Arr-1), β-arrestin-2 (β-Arr-2) or Dab2. The efficiency of siRNAs was analyzed on cell extracts (40 µg of proteins) by SDS-PAGE and western blotting (Fig. S1). Cells were incubated with 500 ng/ml PA63 and 100 ng/ml LF for 1 hour at 4°C and different times at 37°C and cell extracts (40 µg of proteins) were analyzed by SDS-PAGE and western blotting to reveal PA SDS-resistant heptamer (pPA^7mer^) and N-terminus of MEK1 (MEK1 (N)). B: Levels of pPA^7mer^ and full length MEK1 were quantified using the Typhoon scanner and normalized to 1 and 100% respectively at time 0 (1 hour at 4°C). The plot represents the means of 4 independent experiments. Errors represent standard deviations. C: Hela cells were transfected 72 hours with CMG2-V5 and control siRNAs or siRNAs against AP-1, AP-2 or β-arrestin-2 (β-Arr-2). Cell extracts were subsequently analyzed by SDS-PAGE and western blotting to reveal the different forms of PA, CMG2-V5 and N-terminus of MEK1 (MEK1 (N)).

Silencing of Grb2, Dab2 and the µ subunit of the AP-2 complex had no effect on anthrax toxin entry into Hela cells (Fig. 2AB). The lack of involvement of AP-2 was somewhat surprising, raising the possibility that AP2 was insufficiently knocked down to see an effect on endocytosis. We therefore monitored the entry of a second bacterial toxin, namely diphtheria toxin, which has also been shown to enter cells via clathrin-mediated endocytosis [20] as confirmed here (Fig. S2A). Diphtheria toxin is an ADP-ribosylating toxin that modifies elongation factor 2 thus leading to the inhibition of protein synthesis [21]. In contrast to the anthrax toxin, entry of diphtheria toxin was delayed in AP-2, but not AP-1, RNAi treated cells, when compared to cells treated with an irrelevant RNAi (Fig. S2B), indicating that silencing of AP-2 was efficient. It has been previously reported for the EGF-receptor that the temperature at which the ligand is added to the cells affects the apparent AP-2 dependence of the process, i.e. EGF-R endocytosis in AP-2 RNAi treated cells still occurred when the ligand was pre-bound at 4°C, but not when the ligand was added to the cells at 37°C [22]. We therefore repeated our knockdown experiments, omitting the pre-binding of PA63 at 4°C and adding it directly to cells at 37°C. In contrast to what was observed for EGF receptor, we still found that AP-2 depletion had no effect on the formation of pPA^7mer^ (Fig. S3A).

In marked contrast to the knock down of AP-2, silencing the µ subunit of the AP-1 adaptor drastically delayed the formation of the SDS-resistant pPA^7mer^ and LF-mediated MEK1 cleavage in Hela cells Fig. 2AB. This was not due to an off-target effect of the RNAi duplexes, since a similar effect was observed upon silencing of the γ subunit of the AP-1 complex (Fig. S3B). The lack of pPA^7mer^ formation was not due to an effect of AP-1 silencing on endosome acidification–due for example to altered trafficking of vATPase components–since diphtheria toxin, which as the anthrax toxin requires transport to an acidic compartment for cytoplasmic delivery of the enzymatic subunit, retained full activity in AP-1 depleted cells (Fig. S2B). In addition to a requirement for AP-1, we found that silencing β-arrestins strongly delayed formation of pPA^7mer^ Fig. 2AB. Knowing that Hela cells mainly express TEM8, the above experiments indicate that TEM8-mediated uptake depends on AP1 and β-arrestins but not on AP-2. To investigate whether CMG2-mediated uptake had the same requirements, we repeated the above experiments in Hela cells transfected with CMG2. As shown in Fig. 2C, formation of pPA7mer was not detected within the first 45 min when silencing AP-1 or β-arrestin, but occurred normally when silencing AP-2, showing that both receptors have the same adaptor requirements.

**Figure 3:**
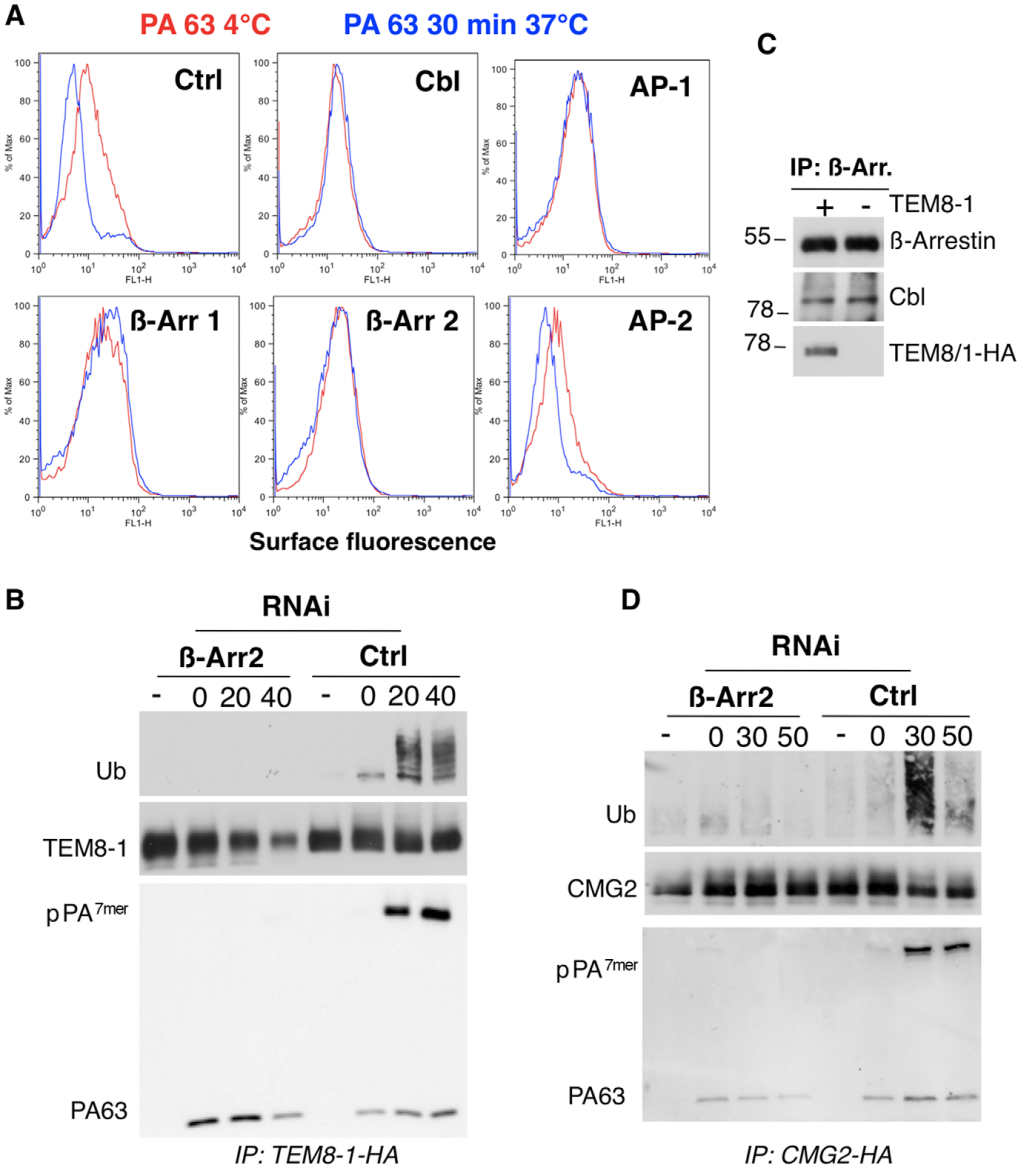
Endocytosis of PA involves the recruitment of Cbl by β-arrestin. A: Hela cells were transfected 72 hours with control siRNAs or siRNAs against Cbl, AP-1, AP-2, β-arrestin-1 (β-Arr-1), or β-arrestin-2 (β-Arr-2). Cells were treated with 1 µg/ml of PA63 for 1 hr at 4°C (red) and 30 min at 37°C (blue) and subsequently submitted to FACS analysis. B: Hela cells were transfected 72 hrs with TEM8/1-HA and with control siRNAs or siRNAs against β-arrestin 2. Cells were then treated or not with 1 µg/ml of PA63 WT for 1 hr at 4°C and different times at 37°C. Immunoprecipitates against TEM8-HA were analyzed by SDS-PAGE and western blotting against Ubiquitin, TEM8-HA and PA. C: Hela cells were transfected or not for 24 hrs with TEM8/1-HA. Immunoprecipitates against β-arrestin were analyzed by SDS-PAGE and western blotting against Cbl, TEM8-HA and β-arrestin. D: Hela cells were transfected 72 hours with human CMG2-HA and with control siRNAs or siRNAs against β-arrestin 2. Cells were then treated or not with 1 µg/ml of PA63 WT for 1 hr at 4°C and different times at 37°C. Immunoprecipitates against CMG2-HA were analyzed by SDS-PAGE and western blotting against Ubiquitin, CMG2-HA and PA.

We next investigated whether the delay in pPA^7mer^ formation observed in Hela cells depleted in AP-1 or β-arrestins was due to an effect on the initial step of uptake from the plasma membrane. We included the Cbl silencing in this analysis since the role of ubiquitination in the initial uptake of plasma membrane proteins is somewhat unclear in mammalian cells [23]. Using our FACS based endocytosis assay, PA was found to remain at the cell surface upon silencing of the µ subunit of AP-1, of β-arrestins as well as of Cbl, while silencing of the µ subunit of AP-2 had no effect (Fig. 3A).

As has been well established, endocytosis of PA requires heptamerization [1],[9]. Therefore the lack of endocytosis of PA upon RNAi silencing of genes could be due either to an effect on heptamerization or on endocytosis itself. Heptameric PA at the cell surface is sensitive to SDS and thus migrates as a monomer on SDS gels. It can however be converted to an SDS resistant form by submitting cell extracts to a pH 4.5 treatment. Using this treatment, we could rule out that silencing of CHC, β-arrestins or AP-1 affected formation of surface PA^7mer^ in Hela cells (Fig. S3C), altogether showing that the endocytosis process itself was affected upon silencing of these genes.

While the role of AP adaptor complexes in recruiting clathrin to cargo molecules has been well established, the role of β-arrestins is less clear [17]. β-arrestins have been proposed to be adaptors either for clathrin or for E3 ubiquitin ligases to the cargo receptor [24]. We therefore investigated whether silencing β-arrestins would affect ubiquitination of TEM8 or CMG2. Two isoforms of TEM8 have been reported, TEM8-1, which has a long 200 amino acid cytoplasmic tail, and TEM8-2 which has a short truncated tail (Fig. S7A). The long form is thought to be the more ubiquitous form. While silencing of AP-1 had no significant effect on TEM8-1 ubiquitination (Fig. S4), silencing β-arrestins strongly diminished ubiquitination (Fig. 3B, note that the apparent lack of ubiquitination in the 40 min is not due to the lower about of TEM8, since even at higher levels of TEM8, as shown in Fig. S4, no ubiquitination signal can be detected upon β-arrestin silencing). That β-arrestins are involved in recruiting Cbl to the anthrax receptors was further supported by immunoprecipitation experiments. Immunoprecipitation of endogenous β-arrestin indeed led to the co-immunoprecipitation of Cbl and of TEM8-1-HA, when transfected (Fig. 3C). Cbl was not detected in immunoprecipitations against an irrelevant protein (not shown). It is worth mentioning that another E3 ligase known to be involved in endocytosis of certain transmembrane proteins such as the epithelial sodium channel [25], namely Nedd4, is not involved in anthrax toxin endocytosis (Fig. S3B), underlying the specificity of the E3 ligase Cbl. We similarly found that silencing of β-arrestin, prevented PA induced ubiquitination of CMG2 upon ectopic expression in Hela cells (Fig. 3D).

### Intracellular delivery of anthrax toxin is actin dependent

To further dissect the mechanisms governing anthrax toxin endocytosis, we investigated the role of actin. It has previously been reported that production of cAMP triggered by anthrax edema toxin is actin dependent [26]. More recently, Mogridge and colleagues have shown that TEM8-1, but not TEM8-2, interacts with actin and have proposed that interaction with actin modulates the affinity for PA, TEM8-2 having a higher affinity for PA than TEM8-1 [27]. During the course of the present studies, Garlick et al. identified a 33 residue stretch in the cytoplasmic tail of TEM8-1 responsible for actin binding and found that a synthetic peptide encompassing this region could trigger actin bundling *in vitro* [27].

**Figure 4:**
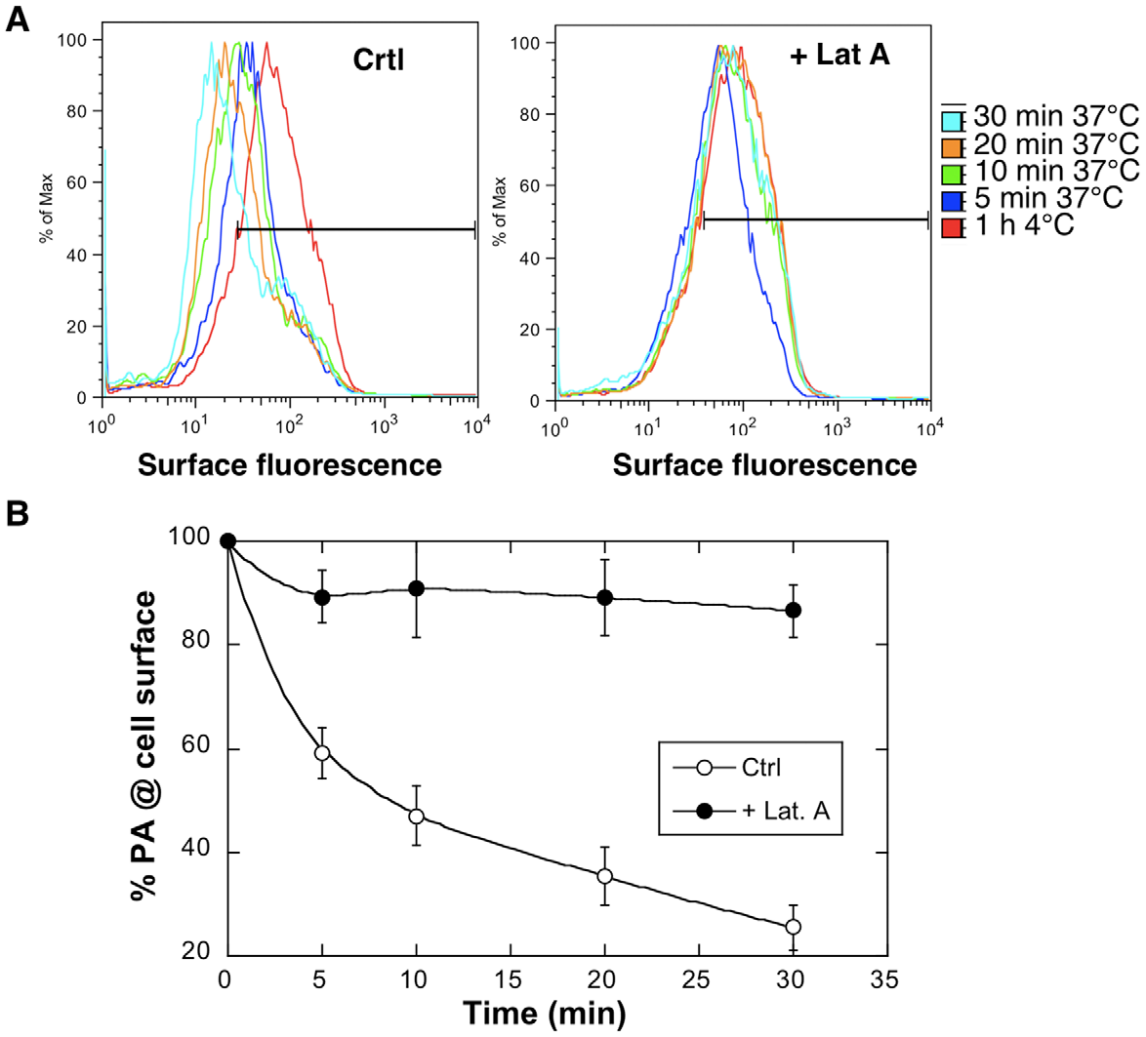
Latrunculin A inhibits anthrax toxin uptake. Hela cells were treated 45 min at 37°C with or without Latrunculin A prior to addition of 1 µg/ml of PA63 for 1 hr at 4°C (red) followed by different incubation times at 37°C. A: Cells were then submitted to FACS analysis. B: The plot represents the mean of the percentage of PA at the cell surface for 4 experiments. Errors represent standard deviations.

**Figure 5:**
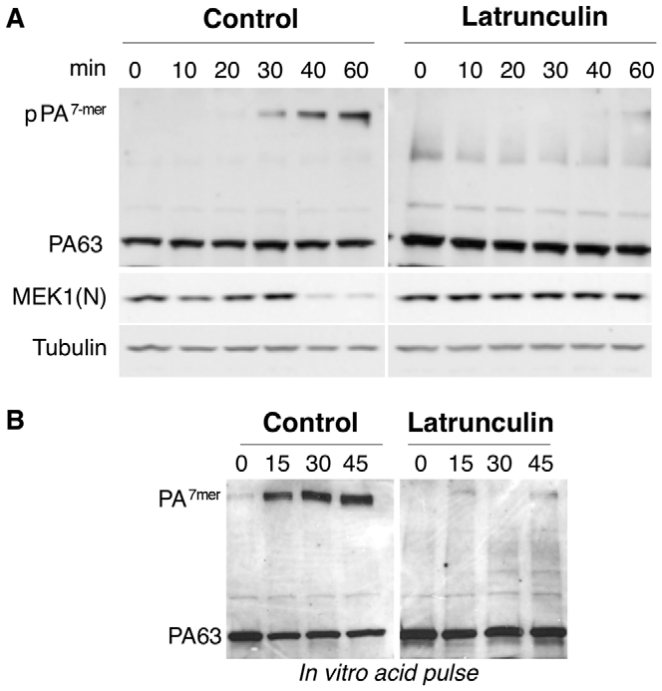
Latrunculin A prevents transport of PA to endosomes and subsequent cleavage of MEK1. A: Hela cells were treated 45 min at 37°C with or without Latrunculin A, prior to the addition of 500 ng/ml of PA63 and 100 ng/ml LF for 1 hr at 4°C followed by different incubation times at 37°C. Cell extracts (40 µg of proteins) were analyzed by SDS-PAGE and western blotting to reveal pPA^7mer^, PA63 and the N-terminus of MEK1 (MEK1 (N)). Tubulin is the equal loading control. B: Cell extracts (40 µg of proteins) described in A were treated 10 min at room temperature with acid buffer pH 4.5 and analyzed by SDS-PAGE and western blotting to reveal the total heptameric PA63 (PA^7mer^) population and PA63 monomer.

We here sought to investigate what the exact role of actin in anthrax toxin endocytosis could be. Using our FACS based PA internalization assay, we monitored the effect of the G-actin sequestering drug Latrunculin A. Whereas PA was rapidly internalized in controls cells, endocytosis was completely blocked in latrunculin treated Hela cells (Fig. 4). This was confirmed by the fact that the SDS-resistant pPA^7mer^ failed to form and MEK1 remained intact (Fig. 5A and Fig. S5).

### Involvement of actin in heptamerization of TEM8-bound PA

As mentioned above, endocytosis of PA requires heptamerization [1]. To test whether latrunculin treatment affected heptamerization of PA at the cell surface, cell extracts were submitted to a pH 4.5 treatment, prior to SDS-PAGE. To our surprise, heptamerization was severely inhibited in latrunculin treated Hela cells, indicating that the drug had blocked the oligomerization process (Fig. 5B). This was not due to an effect of latrunculin on PA heptamerization *per se*, as will become apparent later.

The involvement of actin in PA63 heptamerization at the surface of Hela cells was further confirmed by treating cells with blebbistatin, an inhibitor of myosin II ATPase activity [28]. Although the effect was not as strong as with latrunculin, blebbistatin significantly inhibited both the appearance of endosomal pPA^7mer^ (SDS resistant) and the surface PA^7mer^ (converted to SDS resistant by an acid treatment) (Fig. S6).

**Figure 6:**
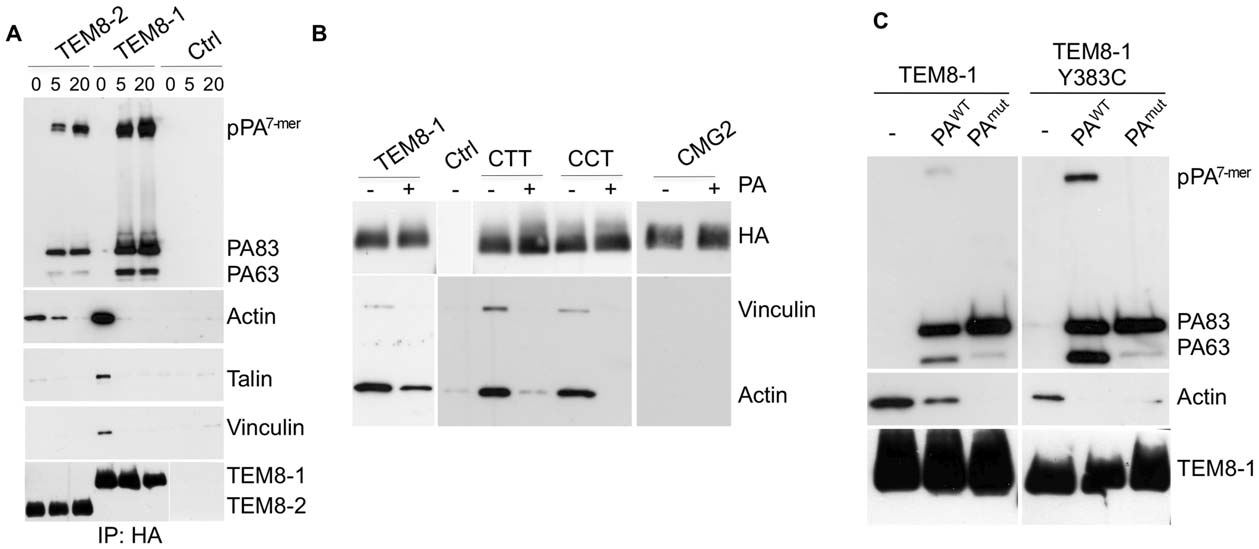
Binding of PA leads to the release of the actin-interacting complex form the cytosolic tail of TEM8. Hela cells were transfected 24 hrs with TEM8/1-HA (ABC) or TEM8/2-HA (B) or CMG2 (B) or constructs CTT (B) or CCT (B) or TEM8/1 Y383C-HA (C). Cells were then treated or not with 1 µg/ml of PA83 WT (ABC) or mutant resistant to furin cleavage (C) for 1 hr at 4°C. A: Cells were subsequently incubated 5 or 20 minutes at 37°C. Immunoprecipitates against TEM8-HA were analyzed by SDS-PAGE and western blotting against Actin, Talin, Vinculin, TEM8-HA and PA. B: Cells were subsequently incubated 10 minutes at 37°C. Immunoprecipitates against HA were analyzed by SDS-PAGE and western blotting against Vinculin, Actin and HA. C: Cells were subsequently incubated 10 minutes at 37°C. Immunoprecipitates against TEM8-HA were analyzed by SDS-PAGE and western blotting against Actin, TEM8-HA and PA.

The above experiments show that the actin cytoskeleton promotes toxin oligomerization in Hela cells. We next wished to confirm the findings of Go et al. on the differential ability of TEM8 isoforms 1 and 2 to interact with actin [29]. Actin could be readily detected upon immuno-precipitation of HA-tagged TEM8-1 expressed in Hela cells. We could also co-immunoprecipitate the actin nucleating protein talin, its interacting protein vinculin (Fig. 6A) and the blebbistatin sensitive myosin II heavy chain 9, MyH9 [30] (Fig. S7B). Since the interaction of TEM8-1 with actin appears to be direct [27], talin, vinculin and myosin II might be involved in regulating the dynamics of the process, as suggested by the here-observed effects of blebbistatin. This will however require further investigation.

In contrast to the observations reported by Go et al. [29], we could also detect actin in TEM8-2-HA immunoprecipitates, albeit at far lower levels than for TEM8-1-HA. In agreement with Go et al. [29], the Y383C mutation, which mimics a mutation found in Hyaline fibromatosis syndrome [31],[32], affected actin binding, but in our hands did not abolish it (Fig. 6C). We were however unable to detect any co-immunoprecipitation of actin with CMG2 (Fig. 6B). This was somewhat surprising considering the high degree of similarity between the cytoplasmic tails of CMG2 and TEM8-1 (Fig. S7A). Since the tail of TEM8-1 is some 50 residues longer than that of CMG2, we generated a truncated TEM8-1 matching the length of CMG2, and found that the ability to immunoprecipitate actin was unaffected (not shown). We also constructed chimeric proteins in which the transmembrane and/or cytosolic tail of CMG2 were replaced by that of TEM8-1 and found that the cytosolic tail of TEM8-1 is sufficient to confer actin-binding ability to the proteins (Fig. 6B). Garlick et al. have narrowed down the actin interacting domain of TEM8-1 with actin to residues 379 to 411 (DASYYGGRGVGGIKRMEVRWG**E**KGSTEEGAKLE) [27], a stretch that is absent in TEM8-2 but fully conserved, with the exception of a single residue (bold underlined), in CMG2. Combined, the observations of Mogridge and ours thus suggest that direct binding of the 379–411 amino acid stretch to actin must be prevented in CMG2, through regions that differ from the tail of TEM8-1 (Fig. S7A). The observation that TEM8-2 can, albeit to a far lesser extend, also interacts with actin suggest that TEM8 might be able to interact with actin by more than one way, possibly directly and indirectly via the interaction with talin, vinculin and myosin II, an interaction that would also be prevented in CMG2.

The effect of latrunculin A on PA heptamerization prompted us to evaluate the effect of PA binding on the interaction of TEM8 with actin. Interestingly, when TEM8-1 (WT or Y383C mutant) was immuno-precipitated from PA83 treated cells, the interaction with actin, talin and vinculin was strongly diminished Fig. 6AC. The decreased interaction with actin did not require the oligomerization of PA63 since it was equally observed when treating cells with a mutant of PA that is resistant to furin cleavage and thus remains monomeric (Fig. 6C) [8]. These observations show that TEM8-1, as well as TEM8-2 albeit to a far lesser extent, interact with the actin cytoskeleton, but that binding of PA83 releases this interaction. These findings provide a mechanistic explanation for the observations of Mogridge and colleagues that the association of TEM8 with the cytoskeleton correlates with weakened binding to PA [27],[29]. They also provide the first evidence that binding of the toxin to TEM8-1 on the outside of the cell leads to a conformational change on the cytosolic side, corresponding to a form of outside-in signaling. Go et al. recently described inside-out signaling where by actin might govern the affinity of TEM8 for its ligand [29]. Thus TEM8 is, as integrins, capable of both inside-out and outside-in signaling.

### Receptor mobility and actin dependence

**Figure 7:**
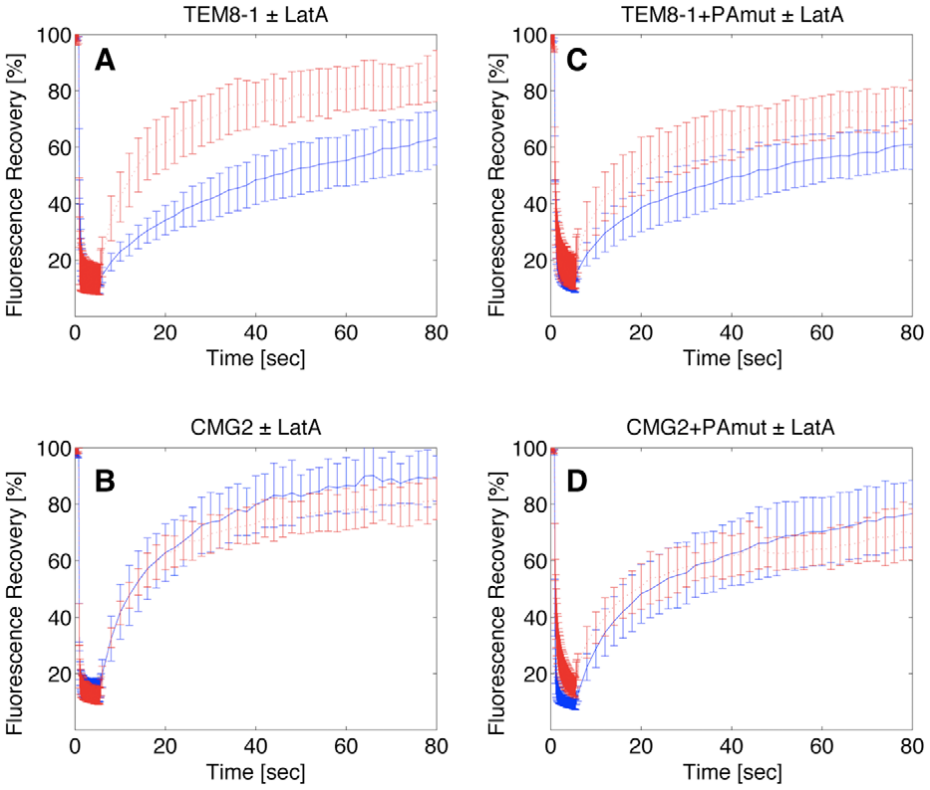
Effect of latrunculin A on the surface mobility of TEM8-1 and CMG2. Hela cells were transfected 48 hrs with TEM8-1-GFP (AC) or CMG2-GFP (BD) and treated (red curves) or not (blue curves) with Latrunculin A. AB: Cells were submitted to FRAP analysis in the absence of toxin treatment. CD: Cells were incubated with 3 µg/ml of mutant PA, resistant to furin cleavage and thus defective in heptamerization, at 4°C for 1 hr and subsequently warmed back to room temperature, in the presence or absence of latrunculin A, for FRAP analysis. Each curve is the average of at least 9 different cells. Error bars represent standard deviations of the mean at each time point.

Using fluorescence recovery after photobleaching (FRAP), Go et al. [29] have shown that the motilities of TEM8-1 and TEM8-2 at the cell surface differ, TEM8-1 being less mobile with a 25% immobile fraction. The difference between the two isoforms was however abolished after latrunculin treatment [29]. We made the same observations after expression of GFP-fusions of these proteins in Hela cells and FRAP analysis (Fig. 7A for TEM8-1; TEM8-2 not shown). More specifically, the recovery half-life (t_1/2_) of TEM8-1 was 27.4±5.4 s in control cells, with an immobile fraction of 30±9%. After latrunculin treatment, the immobile fraction dropped to 16±7% and t_1/2_ decreased to 12.2±4.9 s. We extended these analyses to CMG2. As predicted from the biochemical observations, we found no effect of latrunculin A treatment on the mobility of CMG2 using FRAP: t_1/2_ was 13.3±4.2 s with 9±9% immobile fraction in control cells vs. t_1/2 = _11±4.2 s in latrunculin treated cell, p = 0.21 (Fig. 7B).

FRAP analysis also confirmed the observation that PA binding alleviates the interaction with actin. When cells were incubated with the PA, the latrunculin A dependence of t_1/2_ for TEM8-1 was indeed strongly reduced (Fig. 7C) and in certain experiments even abolished. These experiments were performed using the mutant PA that cannot heptamerize, in order to focus on the effect of toxin binding, without the complications of oligomerization and internalization. The residual effect of latrunculin A could be due to the fact that not all TEM8-1 molecules are occupied by PA, and this may also account for the variability between experiments performed on transiently transfected cells, where expression levels are not tightly controlled. PA binding had not effect on the latrunculin A dependence of CMG2 mobility as expected from the biochemical observations (Fig. 7D).

### Differential receptor-dependent requirement for actin in toxin uptake

**Figure 8:**
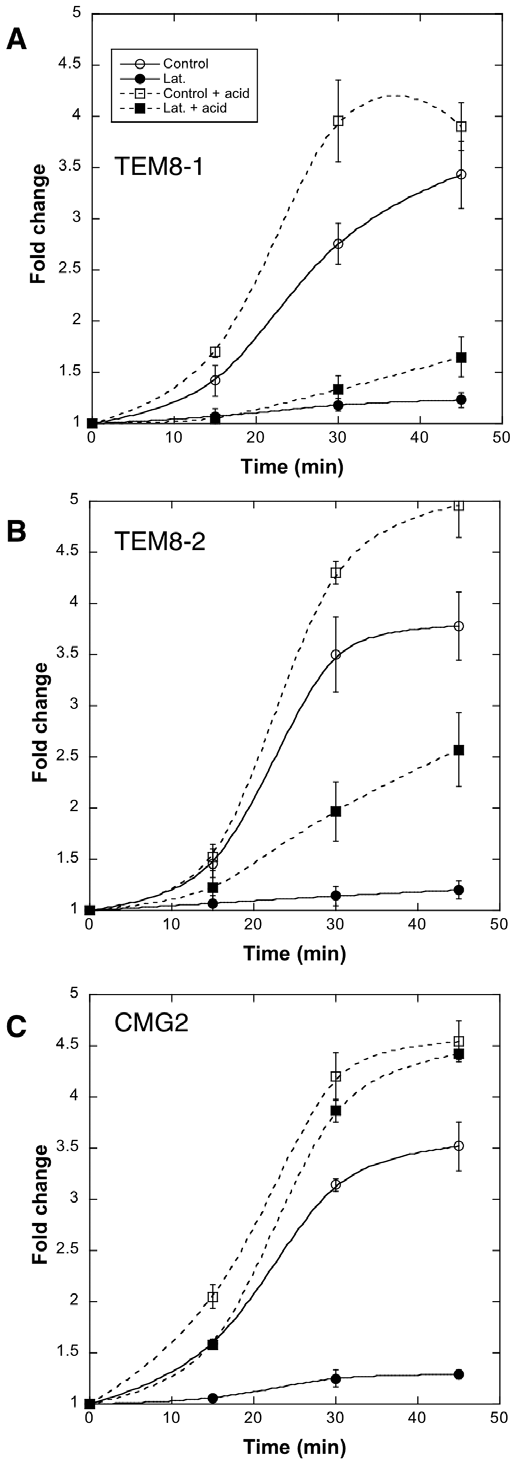
Receptor dependent actin requirements for PA heptamerization and endocytosis. CHO^ΔATR^ cells were transfected 48 hrs with TEM8/1-HA (A) or TEM8/2-HA (B) or CMG2/4-V5 (C) or empty pCDNA3 plasmid (control). Cells were then treated 45 min at 37°C with (filled symbols) or without (open symbols) Latrunculin A, prior to the addition of 500 ng/ml of PA63 and 100 ng/ml LF for 1 hr at 4°C followed by different incubation times at 37°C. Total cell extracts were either analyzed directly by SDS-PAGE and western blotting against PA or first treated 10 min at room temperature with acid buffer to reveal the total PA63 heptameric population (surface + intracellular: dashed lines). PA^7mer^ and pPA^7mer^ levels were quantified using the Typhoon scanner and normalized to 1 at the level at time 0 (1 hour at 4°C). The plot represents the mean of 3 independent experiments. Errors represent standard deviations.

The differential abilities of TEM8-1, TEM8-2 or CMG2 to interact with actin suggested that the requirement for actin in toxin uptake might depend on the receptor used. To test this, we made use of a CHO mutant cell lines that is devoid of anthrax toxin receptors (CHO^ΔATR^) and thus defective in toxin binding [8]. These cells were recomplemented with TEM8-1, TEM8-2 or CMG2 and the formation of the SDS-resistant pPA^7mer^ was monitored. To follow the heptamerization process, we also, in a parallel set of gels, submitted cell extracts to the pH 4.5 treatment mentioned above, that converts all PA^7mer^ to an SDS-resistant form. As expected, recomplementation with TEM8-1 led to the same observation as in HeLa cells: latrunculin A treatment prevented heptamerization of PA, thus even after acid treatment, heptamers could not be detected (Fig. 8A). In cells expressing TEM8-2, formation of pPA^7mer^ was also completely blocked by latrunculin A. When monitoring the heptamerization process however, it became apparent that latrunculin A affected the process, but much less than for TEM8-1 (≈40% residual oligomerization, Fig. 8B). Finally, in cells recomplemented with CMG2, formation of pPA^7mer^ was again drastically affected by latrunculin, but in striking contrast to the observations made for TEM8, surface heptamerization was unaltered (indicating that latrunculin does not inhibit the heptamerization process *per se*) (Fig. 8C). The absence of pPA^7mer^, despite normal heptamerization, indicates that latrunculin A blocked the endocytic process. To test this directly, we monitored endocytosis of PA in cells recomplemented with CMG2-GFP using our FACS assay, and found that indeed latrunculin A blocked endocytosis (Fig. S8).

Altogether these observations show that 1) actin is required for efficient heptamerization of PA when bound to TEM8-1, to a lesser extent when bound to TEM8-2, but not when bound to CMG2; 2) actin is required for the endocytosis of heptameric PA, probably irrespective of the receptor, as observed for CMG2 and TEM8-2.

While actin was shown to play an active role in the formation of endocytic vesicles in yeast [33], the situation is less clear in mammalian cells. To test whether actin is systematically required for clathrin-mediated endocytosis, we investigated the effect of latrunculin on diphtheria toxin entry. As shown in Fig. S2C, latrunculin had no effect on the kinetics of diphtheria mediated ADP-ribosylation of EF-2, illustrating that clathrin-dependent endocytosis can be actin independent.

## Discussion

The present work provides a more complete view of anthrax toxin endocytosis and uncovers an interesting and unexpected role for actin. In the absence of toxin, the two anthrax toxin receptors, TEM8-1 and CMG2, reside in somewhat different environments. TEM8 appears to be actively organized in an actin dependent manner, while CMG2 is either more randomly distributed or organized in an actin independent manner. Following toxin binding, processing by furin and oligomerization, the receptor tail undergoes *src*-dependent phosphorylation [10] and subsequent β-arrestin-dependent Cbl-mediated ubiquitination. Ubiquitination in turn allows endocytosis via a pathway that appears to be independent of AP-2, Eps15, Grb2, Dab2 (this paper) or AP180 (not shown) but requires the multimeric adaptor AP-1, dynamin, clathrin and actin.

### β-arrestins in clathrin-dependent anthrax toxin endocytosis

Our previous findings that anthrax toxin endocytosis is dependent on dynamin, while independent of caveolin [8], pointed towards a requirement for clathrin. We now show that silencing of CHC inhibits endocytosis of the toxin. Clathrin-mediated endocytosis however does not correspond to a unique entry route but encompassed a collection of internalization pathways that are linked by the use of the clathrin coat protein. The last years have indeed revealed a highly flexible system where each membrane cargo protein recruits, via “sorting signals” in its cytoplasmic domain, a specific set of adaptors and accessory proteins that then interact with clathrin [15]. In addition, some “add on” modules [15] maybe allow and modulate interactions with the actin cytoskeleton (see below).

We had previously shown that ubiquitination of anthrax toxin receptors is necessary for the formation of pPA7^mers^ in endosomes [11]. We had however not determined the step at which ubiquitination is required, i.e. removal from the plasma membrane or sorting into intraluminal vesicles of multivesicular endosomes. Here we show that ubiquitination by Cbl is at least required for the initial step of endocytosis, much like what has been observed in yeast. Interestingly, we found that ubiquitination of the receptors depends on β-arrestins. These adaptor molecules have been implicated in the endocytosis of G coupled receptors, as well as single-transmembrane receptors, such as the tyrosine kinase receptor IGF-1R [18], the Drosophila Notch receptor [19] and others (for review see [34]). Since the tails of β-arrestins contain clathrin and AP-2 interaction motifs, they have been proposed to act as adaptors to bring clathrin to the cargo receptor [17]. Recent studies in yeast identified a family of arrestin related proteins–so called ART (arrestin-related trafficking adaptors) proteins–that mediate endocytosis of specific plasma membrane proteins. Instead of acting as an adaptor between the receptor and AP-2, ARTs were shown to act as adaptors for the Nedd4-like E3 ubiquitin ligase Rsp5 [24],[35]. Such an adaptor function of arrestins to recruit ubiquitin ligases has also been proposed for the β2-adrenergic receptor [17]. It is likely that β-arrestin is important for recruitment of RING domain containing ligase Cbl to the cytoplasmic tail of anthrax toxin receptors. β-Arrestin antibodies indeed precipitated both TEM8-1 and Cbl and depletion of β-arrestin prevented the Cbl-mediated ubiquitination of TEM8-1. Combined with the observations in yeast, our findings illustrate that β-arrestins are able to recruit E3 ligases both of RING (here) and HECT (in yeast) domain containing E3 ligases.

### Role of AP-1 in clathrin-dependent anthrax toxin endocytosis

Both TEM8-1 and CMG2 contain two YXXF motifs (F: bulky hydrophobic, http://elm.eu.org/) in their cytoplasmic tails that are potential interaction sites with heterotetrameric clathrin adaptors. The surprise was to find a requirement for AP-1, but not AP-2. The best-described role of AP-1 is in budding of clathrin coated vesicles from the Trans-Golgi network and endosomes [36]. Therefore the apparent requirement of AP-1 in anthrax endocytosis could be due to an indirect effect, such as the surface delivery of a required component. AP-1 silencing however had no effect on the level of the anthrax receptors at the cell surface–toxin binding was unaffected–, nor on endocytosis of diphtheria toxin or acidification of endosomes. Moreover, this is not the first report for a role of AP-1 at the cell surface. AP-1 was found to be required for phagocytosis in macrophages and in Dictyostelium, and was detected on nascent phagosomes [37] possibly to deliver membrane. Similarly a requirement for AP-1, but not AP-2, was found for clathrin-mediated entry of the human bacterial pathogen *Listeria monocytogenes* and AP-1 localized to the entering bacterium [38] again possibly to deliver membrane. Finally, AP-1 was recently found to be able to functionally compensate for AP-2 in mediating the recycling of synaptic vesicles [39]. Although we cannot fully exclude an indirect effect of AP-1 silencing, our data combined with that of recent literature do suggest that AP-1 could play a role in specific types on endocytosis.

### Role of actin in anthrax toxin endocytosis

When analyzing the role of actin in anthrax toxin endocytosis, we found that TEM8-1 driven heptamerization of PA was strongly affected by actin depolymerizing drugs or inhibitors of the myosin II motor. Intriguingly, whereas TEM8-1 was found to interact with actin in control cells, this interaction was lost upon toxin binding. Our interpretation of these findings is that the cortical actin cytoskeleton actively organizes TEM8-1 at the cell surface, in a manner that favors the oligomerization process. Similarly Mayor and coworker recently found that actin actively organizes GPI-anchored proteins into domains [40]. We are not insinuating that GPI-anchored proteins and TEM8-1 reside in similar domains, was it only because GPI-anchored are well established to associate with lipid rafts, which is not the case for TEM8-1 at steady state [8],[11]. The similarities between TEM8-1 and GPI-anchored proteins in terms of actin dependence do however illustrate the capacity of the cortical cytoskeleton to organize protein domains within membranes.

A second surprising observation was that whereas latrunculin led to an increase in the 2 dimensional diffusion coefficient of TEM8-1 in the plasma membrane (FRAP experiments), it inhibited heptamerization. The efficiency of oligomerization depends on the collision probability between receptor bound PA monomers. Therefore, one would expect that increased motility would lead to accelerated oligomerization. This was however not the case, indicating that the actin dependent localization/organization of TEM8-1 on the membrane is more important for efficient oligomerization than the ability of this receptor to rapidly diffuse at the cell surface.

Despite the high degree of similarity between the cytoplasmic tail of CMG2 and that of TEM8, and in particular the conservation of the various potential motifs for binding of actin or actin interacting proteins (multiple potential SH3 binding motifs, profiling binding motifs and possibly a distant WASP interacting motif, Fig. S7A), we could not detect any interaction of CMG2 with actin using three totally independent methods (immunoprecipitation, FRAP and PA heptamerization experiments). Since CMG2 also contains the stretch of residues found by Garlick et al. [27] to mediate direct binding of TEM8-1 to actin, it appears that regions of the cytoplasmic tail of CMG2, that are not conserved in TEM8-1, might be involved in preventing actin binding at steady state, possibly to ensure high ligand binding affinity.

The observation that actin promotes PA heptamerization when the toxin is bound to TEM8-1, but not when bound to CMG2 provides an possible explanation for an issue that has remained mysterious to us. *In vitro* studies have shown that the affinity of PA for the von Willebrand domain of CMG2 is some 3 orders of magnitude higher than that of PA for the von Willebrand domain of TEM8 [3],[41]. Interestingly, Liu et al. recently reported that the apparent functional affinity of PA for its receptor is only 10 times higher for CMG2 than TEM8 [42]. These functional affinities were obtained by performing toxicity tests. It is interesting to note that in these assays performed on living cells, the apparent difference in binding is 100 lower than expected from experiments on purified von Willebrand domains. Finally when analyzing endocytosis of the toxin in cellular systems–i.e. in the absence of competition with an inactive PA mutant, we have not observed any drastic difference in the kinetics of PA heptamerization or of MEK1 cleavage between cells expressing TEM8 vs. CMG2. One speculation to explain these anomalies is that actin promoted oligomerization partially compensates for the lower affinity of PA for the von Willebrand domain of TEM8.

In addition to its role in PA heptamerization, we found that actin is required for the endocytic process, irrespective of the receptor usage. For large cargoes such as the Vesicular Stomatitis virus, it has been proposed that since the transport vesicles is only incompletely coated by clathrin, actin is required to complete the process [43]. Considering the small size of the anthrax toxin, even taking into account heptamerization of PA and binding of EF and LF, partial coating seems somewhat unlikely. In this case, actin could accelerate the pinching off and detachment of the clathrin coated vesicle as observed in yeast [15]. Clarifying the role of actin in clathrin mediated endocytosis in mammalian cells will clearly require further studies using multiple systems and cargoes.

### Concluding remarks

**Figure 9:**
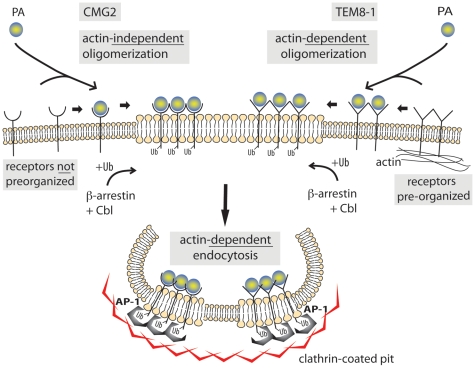
Schematic representation of the endocytosis of the anthrax toxin. TEM8-1 is pre-organized at the cell surface by the cortical actin cytoskeleton while CMG2 is not. Upon PA binding, processing and oligomerization, the toxin receptor complex moves to lipid rafts. There, β-arrestin mediates the recruitment of the E3 ligase Cbl to the cytoplasmic tail of the receptor. The ubiquinated receptor subsequently recruits the heterotetrameric adaptor AP-1 and finally clathrin. Completion of the endocytic process and pinching off of the toxin containing clathrin-coated vesicle requires both actin and dynamin.

Combined with previous reports, our present findings provide the following sequence of events leading to endocytosis of the anthrax toxin (Fig. 9). In the absence of toxin, TEM8-1 interacts with the actin cytoskeleton. This reduces its mobility and leads to some form of surface organization, all of which is not observed for CMG2. Upon secretion by the bacterium and diffusion towards target cells, PA binds to the receptors with an affinity that depends of the receptor identity (higher affinity for CMG2 than TEM8) and on the inside-out signaling, mediated by the actin cytoskeleton, that affects the conformation of the ectodomain of TEM8-1 [29], similar to what is observed for integrins. Upon toxin binding, interaction between TEM8 and the actin cytoskeleton is released. Despite this lost interaction, the actin dependent surface organization/clustering of TEM8-1 favors the heptamerization of PA63. Heptamerization leads to the activation of *src*-like kinases which in turn phosphorylate the cytoplasmic tail of CMG2 [10], as step that is required for the β-arrestin mediated Cbl-dependent ubiquitination of the receptors. The modifications finally allow the recruitment of AP-1 and clathrin, leading to clathrin coated pit formation, which could pinch off and detach through the action of the GTPase dynamin and of actin.

## Materials and Methods

### Cells

Hela cells were grown in complete Modified Eagle's medium (MEM) (Gibco) supplemented with 10% fetal calf serum (FCS), 2 mM L-glutamine, penicillin and streptomycin. The anthrax toxin receptor–deficient CHO (here designated as CHO^ΔATR^) cells were grown in F12 medium as described previously [44],[45].

Stable BHK21-tTA/anti-CHC cells were maintained in Dulbecco's modified Eagle's medium (DMEM) containing 200 ng ml–1 puromycin and 2 µg ml–1 tetracycline [46]. To induce CHC antisense RNA expression, tetracycline was removed from the medium for 48 hours.

### Toxins, antibodies and reagents

Anthrax toxin subunits and diphtheria toxin were a gift from S. Leppla, prepared as described [47], including wild type PA, PA U7 in which the furin cleavage site RKKR is changed to PGG, LF. PA63 corresponds to trypsin-nicked PA83 [8]. Antibodies against anthrax PA were from the Leppla laboratory; the aerolysin mutant (G202C-I445C) named ASSP was produced in our lab as described [48]. The rat anti-mouse CMG2 was generated by genetic immunization with the mouse CMG2 construct (outsourced to Genovac). The antibody against the N-terminal peptide of MEK1 was produced in our laboratory; anti-HA, anti-GFP monoclonals and anti-HA-agarose conjugated beads from Roche (Applied Science, IN); anti-tubulin, anti-AP1, anti-vinculin, anti-talin, anti-MYH9 and anti-β arrestin from Sigma; anti-actin from Millipore; anti-EF2, anti-Cbl, anti-AP2, anti-caveolin, anti-Ubiquitin from Santa Cruz; anti-GRB2 from BD Transduction Laboratories; anti-Dab2 from Abcam laboratories, anti-CHC from Affinity Bioreagents, anti-transferrin receptor from Zymed, anti-caveolin from Santa Cruz, anti-rab 5 was kindly provided by J. Gruenberg. Mouse and rabbit HRP secondary antibodies were from Pierce, IL, rat HRP secondary antibodies were from Sigma and Alexa-conjugated secondary antibodies from Molecular Probes. Latrunculin A was purchased from Invitrogen and used at a final concentration of 0.4 µg/ml for 45 min in medium without serum at 37°C. Blebbistatin was purchased from Sigma and used at a final concentration of 50 µM for 1 hr in medium without serum at 37°C.

### Plasmids and transfection

Human TEM8-HA/1, TEM8-HA/2 and CMG2-4-HA were cloned in pIREShyg2 as described [11]. The human TEM8/1-GFP (isoform 1) and the human TEM8/2-GFP (isoform 2) were cloned in pHS003-EGFP, kindly provided by J. Young. Human TEM8-HA/1 Y383C was generated by mutagenesis using Quickchange (Stratagene) reagent with the following primers: 5′- G GTA GAC GCC TCT TAT TGT GGT GGG AGA GGC GTT GG-3′. The human and mouse CMG2 (isoform 4) gene tagged with a V5 epitope was cloned in pcDNA3.1/V5-HIS-TOPO expression vector. The human CMG2-GFP (isoform 1) was cloned in pHS003-EGFP as described [49].

Synthetic genes were synthesized by Geneart: CTT for extracellular part of CMG2 (amino acids 1 to 318) and transmembrane and cytosolic regions of TEM8 (amino acids 321 to 573), CCT for extracellular and transmembrane regions of CMG2 (amino acids 1 to 341) and cytosolic part of TEM8 (amino acids 344 to 573) and cloned in pIREShyg2 with a HA tag at the C-terminus. The human Eps15 gene was cloned in pEGFP-C2, kindly provided by A. Dautry-Varsat (Pasteur Institute). Plasmids were transfected into Hela cells for 48 or 72 hrs (2 µg cDNA/9.6 cm^2^ plate) using Fugene (Roche Diagnostics Corporation).

### RNAi experiments

siRNA target sequences were the following: human CHC: GGCCCAGGT GGTAATCATTTT, Grb2: AAGTTTGGAAACGATGTGCAG, NEDD4: ATGGAGTTGATTAGATTACAA, Dynamin II: CTGCAGCTCATCTTCTCAAAA, EPS15: GTGGACCAACATAATATTAAA, AP1 µ subunit: AAGGCATCAAGTATCGGAAGA, AP1 γ subunit: AACGAATGTTCGGATGACTTT, AP2 µ subunit: GGAAAACATCAAGAACAATTT, β-arrestin 1: AAAGCCTTCTGCGCGGAGAAT, β-arrestin 2: AAGGACCGCAAAGTGTTTGTG, DAB2: AAGGTTGGCCTTAGTAGTCAA, were purchased from Qiagen. Cbl siRNA was purchased from Santa Cruz (sc-29242). As control siRNA we used the following target sequence of the viral glycoprotein VSV-G: ATTGAACAAACGAAACAAGGA. To do silencing, Hela cells were transfected for 72 hours with 100 pmol/9.2 cm^2^ dish of siRNA using oligofectamine (Invitrogen) transfection reagent.

### Total cell extracts, in vitro acid pulse and western blot analysis

Hela cells were harvested, washed with PBS and homogenized by passage through a 22G injection needle in HB (HB: 2.9 mM imidazole and 250 mM sucrose, pH 7.4) containing the Roche mini tablet protease inhibitors cocktail following manufacturer's instructions. To convert surface PA^7mer^ to an SDS-resistant form, cell extracts were incubated at room temperature for 10 min with 145 mM NaCl and 20 mM MES-Tris, pH 4.5. Protein quantification was done with Pierce BCA kit. Proteins were loaded at 40 µg prot/lane and separated on a 4–20% acrylamide precast Novex gel (Invitrogen) under reducing conditions for PA and native condition for diphtheria toxin and transferred to nitrocellulose membranes (Schleicher and Schuell).

### Immunoprecipitation

For immunoprecipitations, cells were lysed 30 min at 4°C in IP buffer (0.5%NP40, 500 mM NaCl, 500 mM Tris-HCl pH 7.4, 20 mM EDTA, 10 mM NaF, 2 mM benzamidine, and a cocktail of protease inhibitors, Roche), centrifuged 3 min at 2000 g and supernatants were incubated 16 h at 4°C with antibodies and beads.

To follow protein Ubiquitination, 1 mM of NEM was added in the lysis buffer described above.

### Flow cytometric analysis

BHK or Hela cells were incubated one hour at 4°C with 1 ug/ml PA63, washed and incubated different times at 37°C, washed at 4°C and incubated 5 min on ice with cold trypsin. Loosely attached cells were harvested by pipetting and stained for 30 min on ice with anti-PA antibodies, followed stained for 30 min on ice with secondary fluorescent antibodies, washed in PBS+1%FCS and then evaluated on a FACSCalibur™ (Becton Dickinson). FACS data were analyzed using FlowJo software (FlowJo, LLC).

### Fluorescence recovery after photobleaching (FRAP)

HeLa cells were seeded and transfected with GFP-tagged receptors in 35 mm glass bottom dishes (MatTek). Samples were analyzed on a Leica SP2 confocal scanning microscope using a 63x oil immersion objective. After 10 scans of a chosen region a rectangle of 1×3 µm on the edge of a cell was irreversibly bleached using the full power of the 488, 458 and 476 nm laser lines. The bleaching resulted in a ∼80% depletion of fluorescence. Recovery of fluorescence was monitored over 80 seconds with a 2 second interval by scanning the region with a low 488 nm laser power to minimize photobleaching during sampling. The fluorescence of the bleached area was normalized at each time point using a non-bleached control area. Recovery kinetics were fitted to an exponential function.

## Supporting Information

Figure S1 Endocytosis of the anthrax toxin is clathrin mediated. A: The efficiency of the different siRNAs used in the study was analyzed on cell extracts (40 µg of proteins) by SDS-PAGE and western blotting. B: Stable BHK21-tTA/anti-CHC cells were maintained in 2 µg/ml tetracycline (control cells). To induce CHC antisense RNA expression (CHC AS), tetracycline was removed from the medium for 48 hrs. Control and CHC AS cells were treated with 1 µg/ml of PA63 for 1 hr at 4°C (red) and 5 min at 37°C (green). Cells were prepared for FACS analysis. C: Hela cells were transfected 72 hrs with human CMG2-V5 and with control siRNAs or siRNAs against CHC and incubated with 500 ng/ml PA63 for 1 hr at 4°C and different times at 37°C. Immunoprecipitation was performed against CMG2-V5 and samples were analyzed by SDS-PAGE and western blotting to reveal the different forms of PA.
(0.27 MB PDF)

Figure S2 Diphtheria toxin enters cells via a clathrin and AP-2 dependent route that does not require actin. A-B: Hela cells were or not transfected for 72 hrs with the pSuper vector containing the human CHC (A) or with RNAi oligonucleotide control siRNAs or siRNAs against the µ chains of AP-1 or AP-2 (B). Cells were incubated with 500 ng/ml of trypsin-nicked DT different times at 37°C in serum free medium and extracts (40 µg of proteins) were analyzed by Native or SDS-PAGE and western blotting to reveal EF2. C: Hela cells were treated 45 min at 37°C or not with Latrunculin A. Cells were then treated in the presence or absence of Latrunculin A with 500 ng/ml of trypsin-nicked DT different times at 37°C in serum free medium. Cell extracts were prepared and the modification of EF2 analyzed on native or SDS-PAGE (40 µg of proteins).
(0.48 MB PDF)

Figure S3 Endocytosis of the anthrax toxin depends on AP1 but not Nedd4 and AP2. A: Hela cells were transfected 72 hrs with control siRNAs or siRNAs against µ chains of AP-1 or AP-2. Cells were incubated directly at 37°C with 500 ng/ml PA63 and 100 ng/ml LF for different times. 40 µg of proteins were analyzed by SDS-PAGE and western blotting to reveal PA63 and SDS-resistant heptamer (pPA^7mer^). B: Hela cells were transfected 72 hrs with control siRNAs or siRNAs against Nedd4, µ chains of AP-1 or γ chains of AP-1. Cells were incubated with 500 ng/ml PA63 and 100 ng/ml LF for 1 hour at 4°C and different times at 37°C. 40 µg of proteins were analyzed by SDS-PAGE and western blotting to reveal PA63 and SDS-resistant heptamer (pPA^7mer^). C: Hela cells were transfected 72 hrs with control siRNAs or siRNAs against CHC, µ chains of AP-1 or β-arrestin1 (β-Arr1). Cells were incubated with 500 ng/ml PA63 and 100 ng/ml LF for 1 hour at 4°C and different times at 37°C and cell extracts were submitted to a pH 4.5 treatment to convert all heptameric PA to an SDS-resistant form detectable by SDS PAGE. 40 µg of proteins were analyzed by SDS-PAGE and western blotting to reveal PA.
(0.60 MB PDF)

Figure S4 β-arrestin affects ubiquitination of TEM8-1. Hela cells were transfected 72 hrs with control siRNAs or siRNAs against AP-1 and β-arrestin-1 (β-Arr-1) and with TEM8/1-HA. Cells were then treated or not with 1 µg/ml of PA63 WT for 1 hr at 4°C and different times at 37°C. Immunoprecipitates against TEM8-HA were analyzed by SDS-PAGE and western blotting against Ubiquitin, TEM8-HA and PA.
(0.38 MB PDF)

Figure S5 Latrunculin A prevents transport of PA to endosomes and subsequent cleavage of MEK1. Hela cells were treated 45 min at 37°C with or without Latrunculin A, prior to the addition of 500 ng/ml of PA63 and 100 ng/ml LF for 1 hr at 4°C followed by different incubation times at 37°C. Cell extracts (40 µg of proteins) were analyzed by SDS-PAGE and western blotting to reveal pPA^7mer^, PA63 and the N-terminus of MEK1 (MEK1(N)) (see main Fig. 6) Levels of pPA^7mer^ and full length MEK1 were quantified using the Typhoon scanner and normalized to 1 and 100% respectively at time 0 (1 hour at 4°C). The plot represents the means of 4 independent experiments. Errors represent standard deviations.
(0.40 MB PDF)

Figure S6 The myosin II inhibitor blebbistatin inhibits PA heptamerization and endocytosis in Hela cells. A-C: Hela cells were treated 45 min at 37°C with or without blebbistatin, prior to the addition of 500 ng/ml of PA63 and 100 ng/ml LF for 1 hr at 4°C followed by different incubation times at 37°C. A: Cell extracts (40 µg of proteins) were analyzed by SDS-PAGE and western blotting to reveal pPA^7mer^ and PA63. B: Levels of pPA^7mer^ and N-terminus of MEK1 (MEK1(N)). Levels of pPA^7mer^ and full length MEK1 were quantified using the Typhoon scanner and normalized to 1 and 100% respectively at time 0 (1 hour at 4°C). The plot represents the means of 4 independent experiments. Errors represent standard deviations. C: Cell extracts obtained in A were submitted to a pH 4.5 treatment to convert all heptameric PA63 to an SDS-resistant form prior to SDS-PAGE and western blotting against PA.
(0.36 MB PDF)

Figure S7 TEM8-1 interacts with talin, vinculin and myosin II. A: Alignment of the cytoplasmic tails of human TEM8 isoforms 1 and 2 and human CMG2 isoform 4 using the SIM software of the EXPASY server (www.expasy.ch). Regions of identity are shown in yellow. B: Hela cells were transfected 24 hrs with TEM8/1-HA or TEM8/2-HA. Immunoprecipitates against TEM8-HA were analyzed by SDS-PAGE and western blotting against Talin, Vinculin, TEM8-HA and the blebbistatin sensitive myosin II heavy chain 9, MyH9. C: Hela cells were transfected 24 hrs with TEM8/1-HA. Cells were treated 45 min at 37°C with or without Latrunculin A, prior to the addition or not of 500 ng/ml of PA63 for 1 hr at 4°C and 10 minutes at 37°C. Cells were solubilized in 1% Triton-X-100 at 4°C, loaded at the bottom of an Optiprep gradient, and 6 fractions were collected from the top and analyzed by SDS-PAGE and western blotting against Tem8-HA, PA, Transferrin Receptor (Trf-R) and Caveolin 1 (Cav-1).
(0.24 MB PDF)

Figure S8 CMG2 mediated PA endocytosis is latrunculin dependent. CHO^ΔATR^ cells were transfected 48 hrs with CMG2/1-GFP. Cells were treated 45 min at 37°C or not with Latrunculin A in serum free medium. Cells were then treated in the presence or absence of Latrunculin A with 1 ug/ml of PA63 for 1 hr at 4°C (red) and incubated 20 minutes at 37°C (blue), and subsequently submitted to FACS analysis. Only GFP positive cells were gated.
(0.21 MB PDF)
