# Supplementary figures and images for: Endocytosis of the Anthrax Toxin Is Mediated by Clathrin, Actin and Unconventional Adaptors
Source: PLoS Pathog. 2010 Mar 5;6(3):e1000792. doi: 10.1371/journal.ppat.1000792 (PMC2832758; doi:10.1371/journal.ppat.1000792)

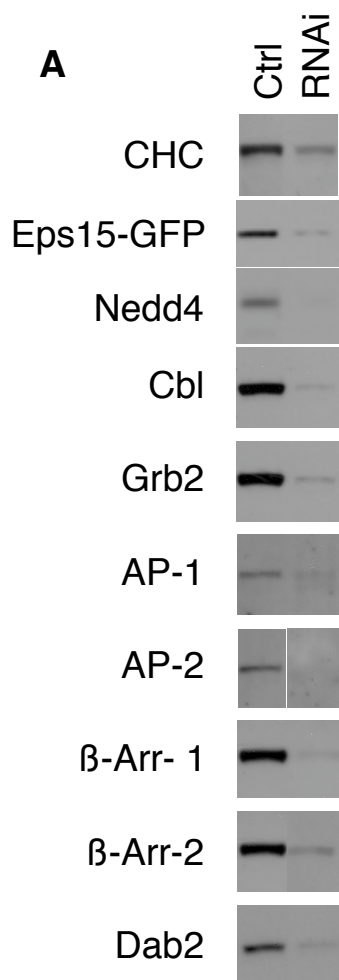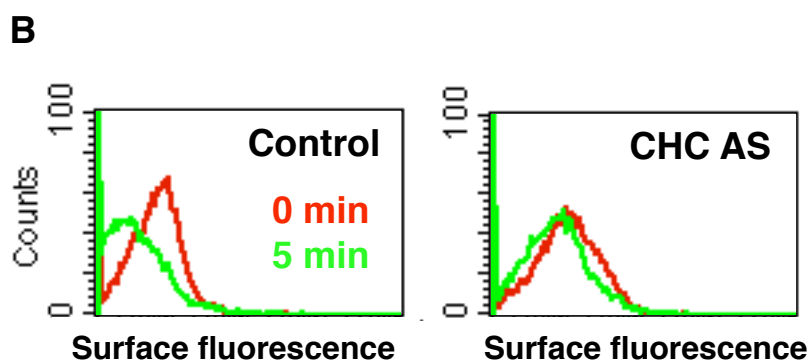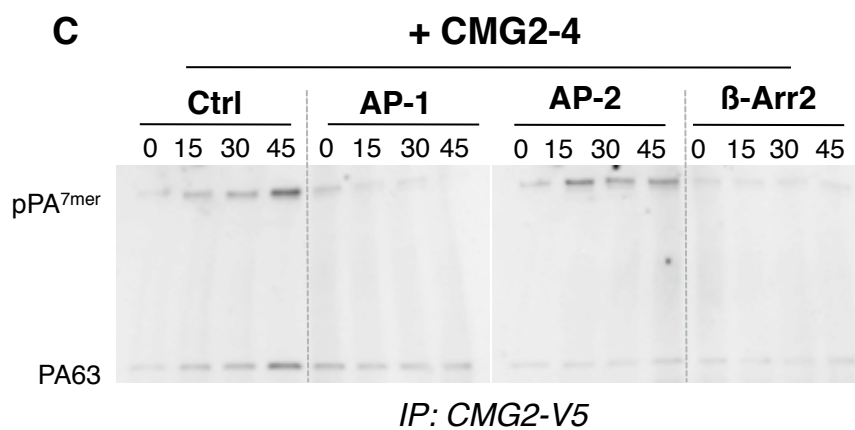

Supplement: Figure S1 — Endocytosis of the anthrax toxin is clathrin mediated. A: The efficiency of the different siRNAs used in the study was analyzed on cell extracts (40 µg of proteins) by SDS-PAGE and western blotting. B: Stable BHK21-tTA/anti-CHC cells were maintained in 2 µg/ml tetracycline (control cells). To induce CHC antisense RNA expression (CHC AS), tetracycline was removed from the medium for 48 hrs. Control and CHC AS cells were treated with 1 µg/ml of PA63 for 1 hr at 4°C (red) and 5 min at 37°C (green). Cells were prepared for FACS analysis. C: Hela cells were transfected 72 hrs with human CMG2-V5 and with control siRNAs or siRNAs against CHC and incubated with 500 ng/ml PA63 for 1 hr at 4°C and different times at 37°C. Immunoprecipitation was performed against CMG2-V5 and samples were analyzed by SDS-PAGE and western blotting to reveal the different forms of PA. (0.27 MB PDF) [file ppat.1000792.s001.pdf]

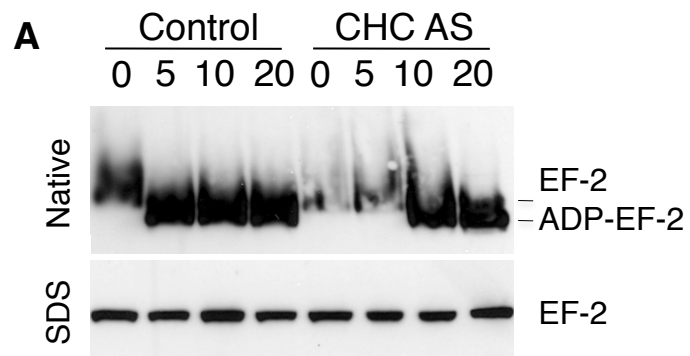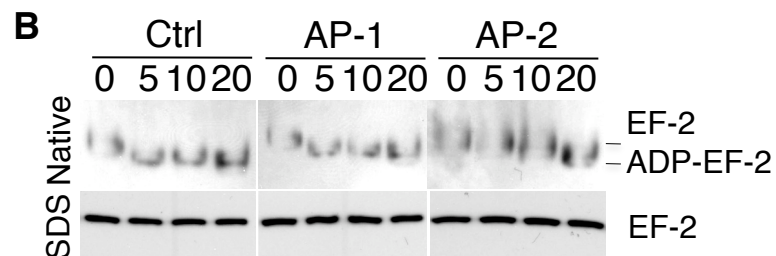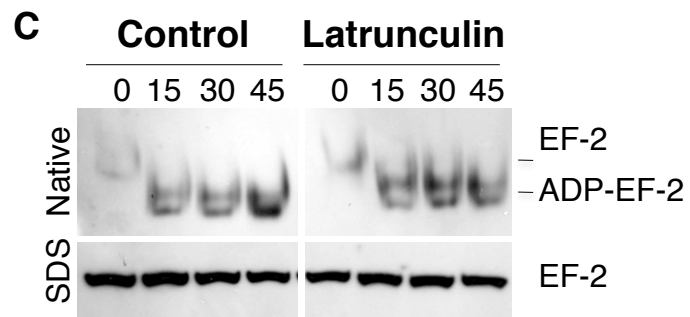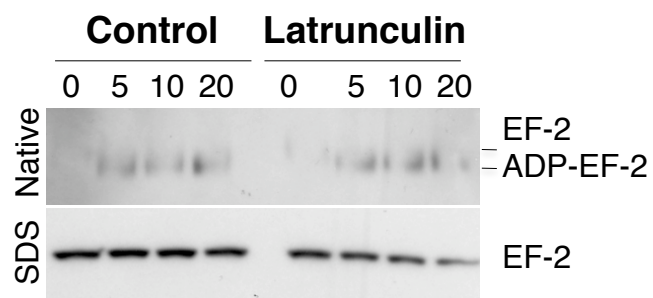

Supplement: Figure S2 — Diphtheria toxin enters cells via a clathrin and AP-2 dependent route that does not require actin. A-B: Hela cells were or not transfected for 72 hrs with the pSuper vector containing the human CHC (A) or with RNAi oligonucleotide control siRNAs or siRNAs against the µ chains of AP-1 or AP-2 (B). Cells were incubated with 500 ng/ml of trypsin-nicked DT different times at 37°C in serum free medium and extracts (40 µg of proteins) were analyzed by Native or SDS-PAGE and western blotting to reveal EF2. C: Hela cells were treated 45 min at 37°C or not with Latrunculin A. Cells were then treated in the presence or absence of Latrunculin A with 500 ng/ml of trypsin-nicked DT different times at 37°C in serum free medium. Cell extracts were prepared and the modification of EF2 analyzed on native or SDS-PAGE (40 µg of proteins). (0.48 MB PDF) [file ppat.1000792.s002.pdf]

**A**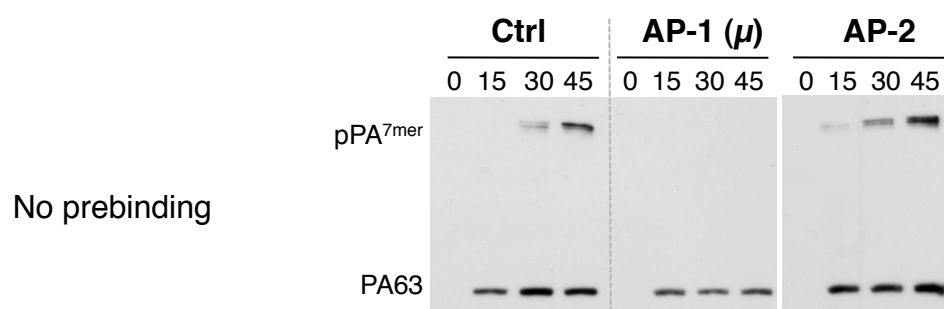**B**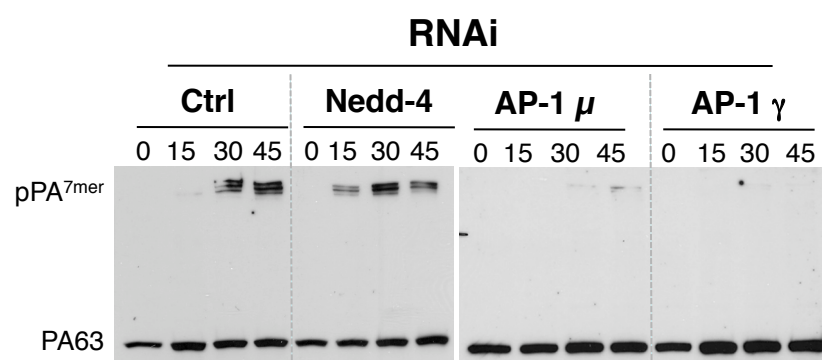**C**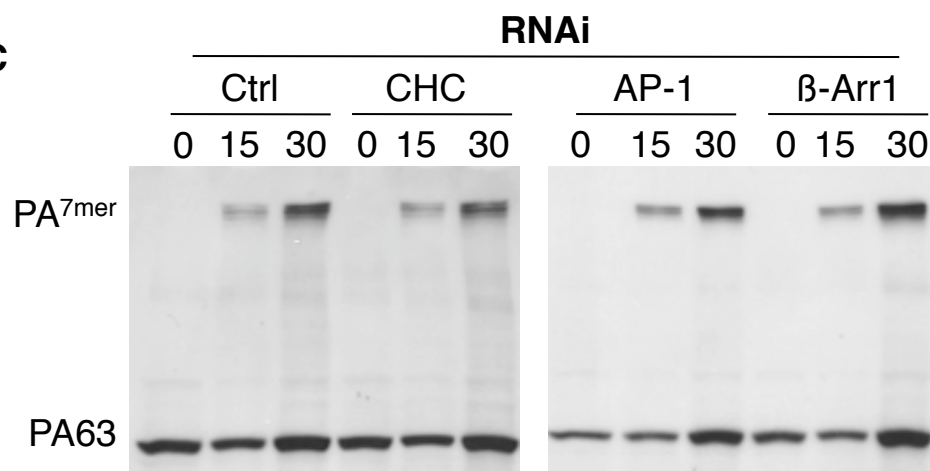

*In vitro acid pulse*

Supplement: Figure S3 — Endocytosis of the anthrax toxin depends on AP1 but not Nedd4 and AP2. A: Hela cells were transfected 72 hrs with control siRNAs or siRNAs against µ chains of AP-1 or AP-2. Cells were incubated directly at 37°C with 500 ng/ml PA63 and 100 ng/ml LF for different times. 40 µg of proteins were analyzed by SDS-PAGE and western blotting to reveal PA63 and SDS-resistant heptamer (pPA7mer). B: Hela cells were transfected 72 hrs with control siRNAs or siRNAs against Nedd4, µ chains of AP-1 or γ chains of AP-1. Cells were incubated with 500 ng/ml PA63 and 100 ng/ml LF for 1 hour at 4°C and different times at 37°C. 40 µg of proteins were analyzed by SDS-PAGE and western blotting to reveal PA63 and SDS-resistant heptamer (pPA7mer). C: Hela cells were transfected 72 hrs with control siRNAs or siRNAs against CHC, µ chains of AP-1 or β-arrestin1 (β-Arr1). Cells were incubated with 500 ng/ml PA63 and 100 ng/ml LF for 1 hour at 4°C and different times at 37°C and cell extracts were submitted to a pH 4.5 treatment to convert all heptameric PA to an SDS-resistant form detectable by SDS PAGE. 40 µg of proteins were analyzed by SDS-PAGE and western blotting to reveal PA. (0.60 MB PDF) [file ppat.1000792.s003.pdf]

## RNAi

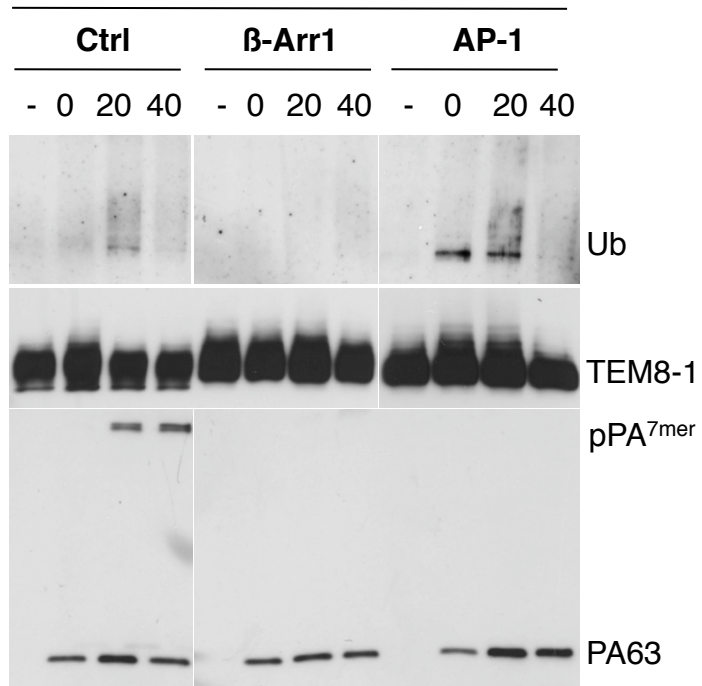

Supplement: Figure S4 — β-arrestin affects ubiquitination of TEM8-1. Hela cells were transfected 72 hrs with control siRNAs or siRNAs against AP-1 and β-arrestin-1 (β-Arr-1) and with TEM8/1-HA. Cells were then treated or not with 1 µg/ml of PA63 WT for 1 hr at 4°C and different times at 37°C. Immunoprecipitates against TEM8-HA were analyzed by SDS-PAGE and western blotting against Ubiquitin, TEM8-HA and PA. (0.38 MB PDF) [file ppat.1000792.s004.pdf]

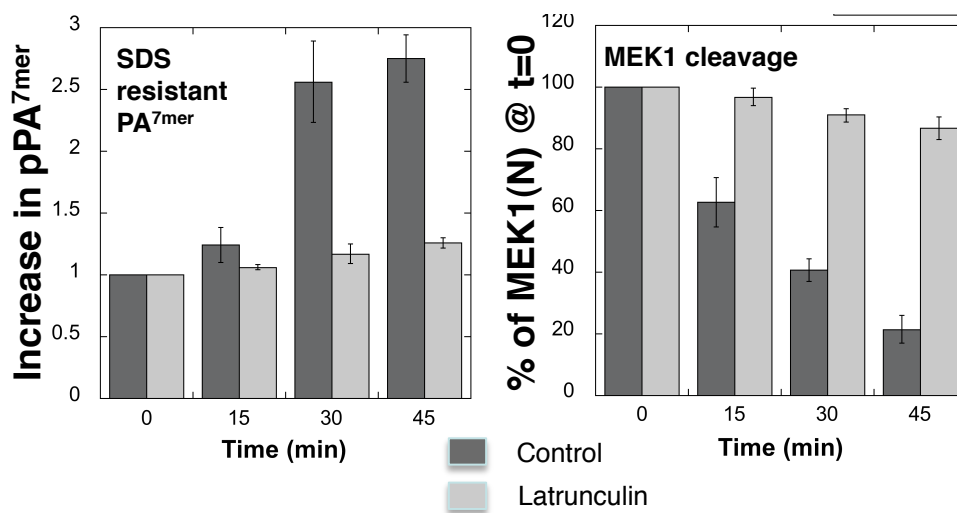

Supplement: Figure S5 — Latrunculin A prevents transport of PA to endosomes and subsequent cleavage of MEK1. Hela cells were treated 45 min at 37°C with or without Latrunculin A, prior to the addition of 500 ng/ml of PA63 and 100 ng/ml LF for 1 hr at 4°C followed by different incubation times at 37°C. Cell extracts (40 µg of proteins) were analyzed by SDS-PAGE and western blotting to reveal pPA7mer, PA63 and the N-terminus of MEK1 (MEK1(N)) (see main Fig. 6) Levels of pPA7mer and full length MEK1 were quantified using the Typhoon scanner and normalized to 1 and 100% respectively at time 0 (1 hour at 4°C). The plot represents the means of 4 independent experiments. Errors represent standard deviations. (0.40 MB PDF) [file ppat.1000792.s005.pdf]

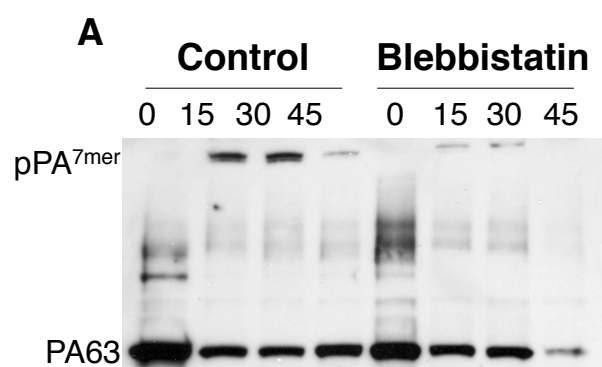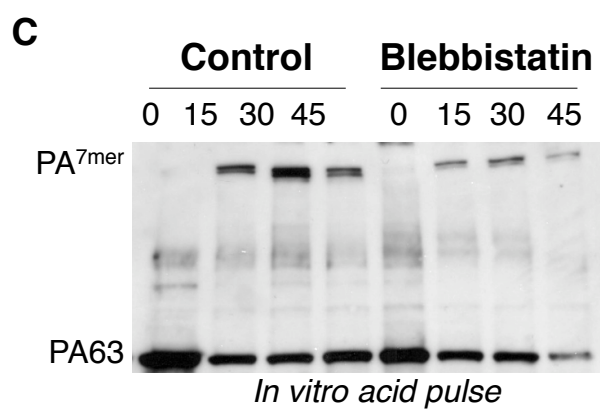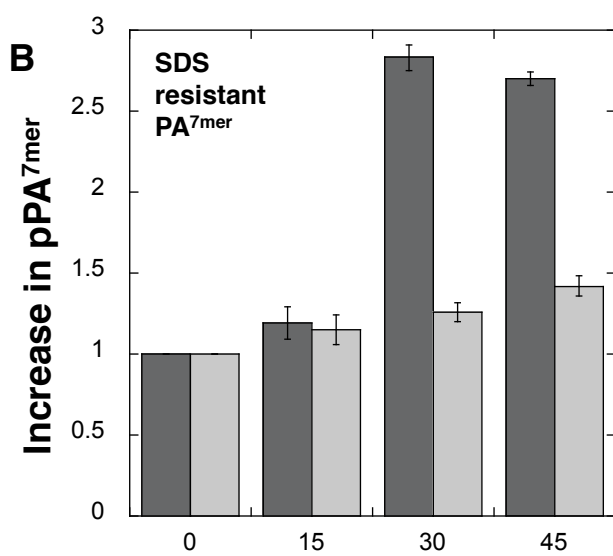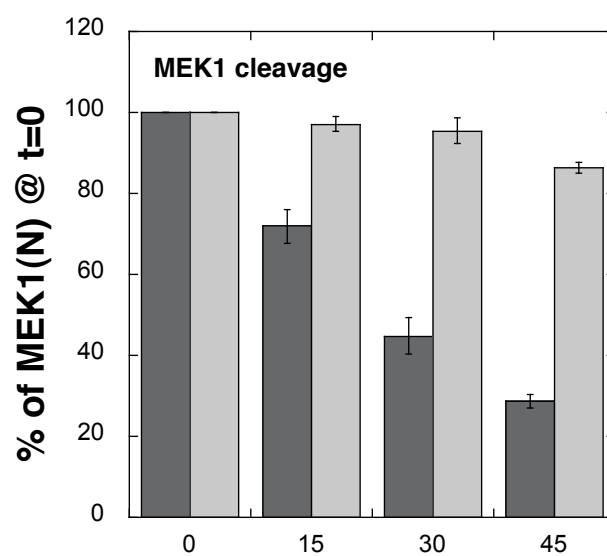

Control  
Blebbistatin

Supplement: Figure S6 — The myosin II inhibitor blebbistatin inhibits PA heptamerization and endocytosis in Hela cells. A-C: Hela cells were treated 45 min at 37°C with or without blebbistatin, prior to the addition of 500 ng/ml of PA63 and 100 ng/ml LF for 1 hr at 4°C followed by different incubation times at 37°C. A: Cell extracts (40 µg of proteins) were analyzed by SDS-PAGE and western blotting to reveal pPA7mer and PA63. B: Levels of pPA7mer and N-terminus of MEK1 (MEK1(N)). Levels of pPA7mer and full length MEK1 were quantified using the Typhoon scanner and normalized to 1 and 100% respectively at time 0 (1 hour at 4°C). The plot represents the means of 4 independent experiments. Errors represent standard deviations. C: Cell extracts obtained in A were submitted to a pH 4.5 treatment to convert all heptameric PA63 to an SDS-resistant form prior to SDS-PAGE and western blotting against PA. (0.36 MB PDF) [file ppat.1000792.s006.pdf]

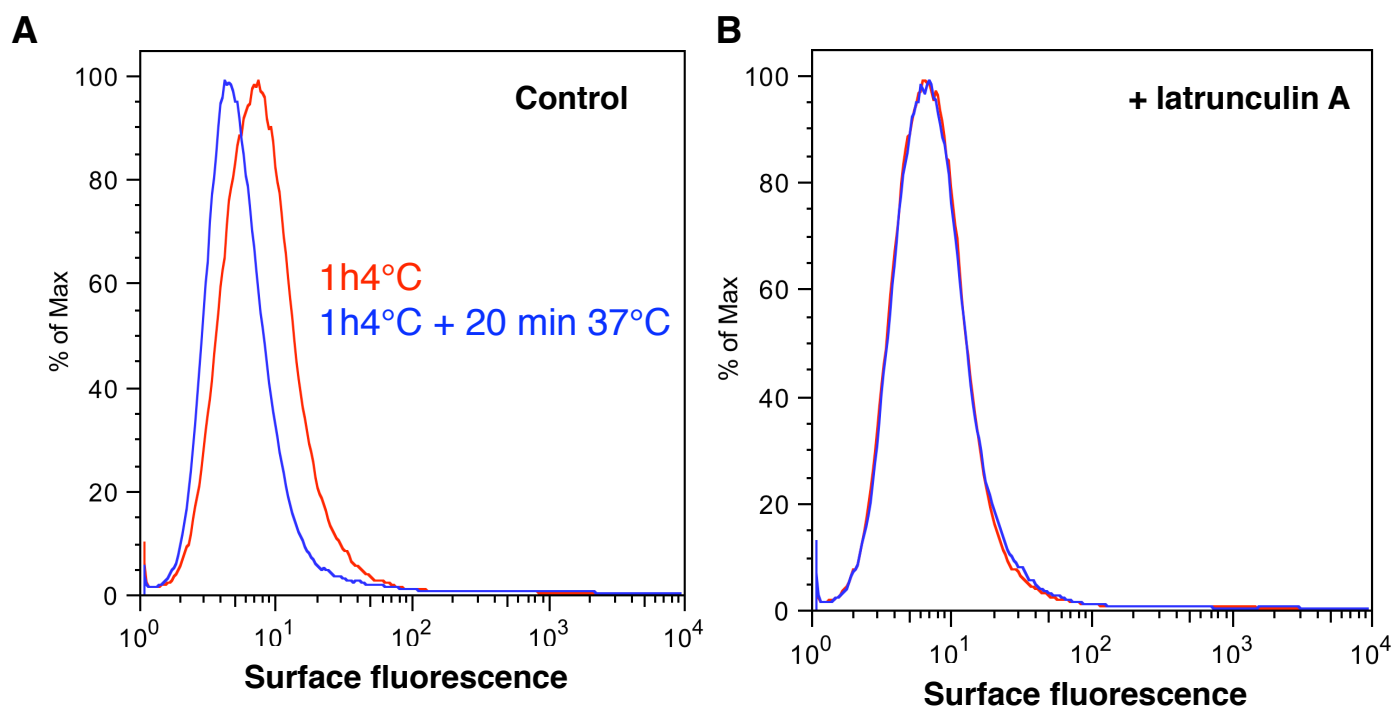

CHO-ΔATR cells expressing CMG2-GFP

Supplement: Figure S8 — CMG2 mediated PA endocytosis is latrunculin dependent. CHOΔATR cells were transfected 48 hrs with CMG2/1-GFP. Cells were treated 45 min at 37°C or not with Latrunculin A in serum free medium. Cells were then treated in the presence or absence of Latrunculin A with 1 ug/ml of PA63 for 1 hr at 4°C (red) and incubated 20 minutes at 37°C (blue), and subsequently submitted to FACS analysis. Only GFP positive cells were gated. (0.21 MB PDF) [file ppat.1000792.s008.pdf]
