# Supplementary material for: Endocytosis of the Anthrax Toxin Is Mediated by Clathrin, Actin and Unconventional Adaptors
Source: PLoS Pathog. 2010 Mar 5;6(3):e1000792. doi: 10.1371/journal.ppat.1000792 (PMC2832758; doi:10.1371/journal.ppat.1000792)

A

```
Tem8-1 PLCCTVIIKEVPPPPAEESEEEDDDGLPKKKWPTVDASYYGGRGVGGIKRMEVRWG
Tem8-2 PLCCTVIIKEVPPPPAEESEENKIK
Cmg2-4 PLCCKVVIKDPPPPPPPAPKEEEEEPLPTKKWPTVDASYYGGRGVGGIKRMEVRWG

Tem8-1 EKGSTEEGAKLEKAKNARVKMPEQEYEFPEPRNLNNNMRRPSSPRKWYSPIKGKLD
Cmg2-4 DKGSTEEGARLEKAKNAVVKIPEETEEPIRPRPPRPKPTHQPPQTKWYTPIKGRLD

Tem8-1 ALWVLLRKGYDRVSVMRPQPGDTGRCINFTRVKNNQPAKYPLNNAYHTSSPPPAPI
Cmg2-4 ALWALLRRQYDRVSLMRPQEGDEV CIWECIEKELTA

Tem8-1 YTPPPPAPHCPPPPPSAPTPPIPSPPSTLPPPPQAPPPNRAPPPSRPPPPRPSV
```

B

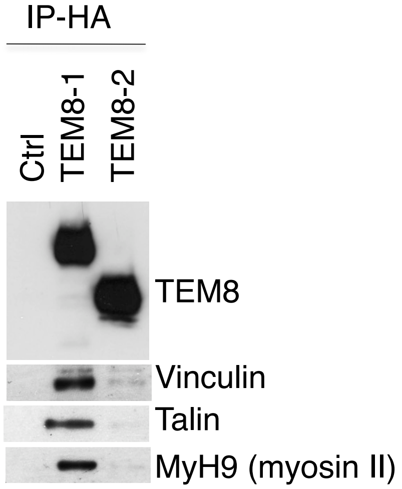

Supplement: Figure S7 — TEM8-1 interacts with talin, vinculin and myosin II. A: Alignment of the cytoplasmic tails of human TEM8 isoforms 1 and 2 and human CMG2 isoform 4 using the SIM software of the EXPASY server (www.expasy.ch). Regions of identity are shown in yellow. B: Hela cells were transfected 24 hrs with TEM8/1-HA or TEM8/2-HA. Immunoprecipitates against TEM8-HA were analyzed by SDS-PAGE and western blotting against Talin, Vinculin, TEM8-HA and the blebbistatin sensitive myosin II heavy chain 9, MyH9. C: Hela cells were transfected 24 hrs with TEM8/1-HA. Cells were treated 45 min at 37°C with or without Latrunculin A, prior to the addition or not of 500 ng/ml of PA63 for 1 hr at 4°C and 10 minutes at 37°C. Cells were solubilized in 1% Triton-X-100 at 4°C, loaded at the bottom of an Optiprep gradient, and 6 fractions were collected from the top and analyzed by SDS-PAGE and western blotting against Tem8-HA, PA, Transferrin Receptor (Trf-R) and Caveolin 1 (Cav-1). (0.24 MB PDF) [file ppat.1000792.s007.pdf]
